# TarKG: a comprehensive biomedical knowledge graph for target discovery

**DOI:** 10.1093/bioinformatics/btae598

**Published:** 2024-10-11

**Authors:** Cong Zhou, Chui-Pu Cai, Xiao-Tian Huang, Song Wu, Jun-Lin Yu, Jing-Wei Wu, Jian-Song Fang, Guo-Bo Li

**Affiliations:** Key Laboratory of Drug-Targeting and Drug Delivery System of the Education Ministry and Sichuan Province, Department of Medicinal Chemistry, West China School of Pharmacy, Sichuan University, Chengdu 610041, China; Division of Data Intelligence, Department of Computer Science, Shantou University, Shantou 515063, China; Key Laboratory of Drug-Targeting and Drug Delivery System of the Education Ministry and Sichuan Province, Department of Medicinal Chemistry, West China School of Pharmacy, Sichuan University, Chengdu 610041, China; Key Laboratory of Drug-Targeting and Drug Delivery System of the Education Ministry and Sichuan Province, Department of Medicinal Chemistry, West China School of Pharmacy, Sichuan University, Chengdu 610041, China; Key Laboratory of Drug-Targeting and Drug Delivery System of the Education Ministry and Sichuan Province, Department of Medicinal Chemistry, West China School of Pharmacy, Sichuan University, Chengdu 610041, China; Key Laboratory of Drug-Targeting and Drug Delivery System of the Education Ministry and Sichuan Province, Department of Medicinal Chemistry, West China School of Pharmacy, Sichuan University, Chengdu 610041, China; Science and Technology Innovation Center, Guangzhou University of Chinese Medicine, Guangzhou 510405, China; Key Laboratory of Drug-Targeting and Drug Delivery System of the Education Ministry and Sichuan Province, Department of Medicinal Chemistry, West China School of Pharmacy, Sichuan University, Chengdu 610041, China

## Abstract

**Motivation:**

Target discovery is a crucial step in drug development, as it directly affects the success rate of clinical trials. Knowledge graphs (KGs) offer unique advantages in processing complex biological data and inferring new relationships. Existing biomedical KGs primarily focus on tasks such as drug repositioning and drug–target interactions, leaving a gap in the construction of KGs tailored for target discovery.

**Results:**

We established a comprehensive biomedical KG focusing on target discovery, termed TarKG, by integrating seven existing biomedical KGs, nine public databases, and traditional Chinese medicine knowledge databases. TarKG consists of 1 143 313 entities and 32 806 467 relations across 15 entity categories and 171 relation types, all centered around 3 core entity types: Disease, Gene, and Compound. TarKG provides specialized knowledges for the core entities including chemical structures, protein sequences, or text descriptions. By using different KG embedding algorithms, we assessed the knowledge completion capabilities of TarKG, particularly for disease–target link prediction. In case studies, we further examined TarKG’s ability to predict potential protein targets for Alzheimer’s disease (AD) and to identify diseases potentially associated with the metallo-deubiquitinase CSN5, using literature analysis for validation. Furthermore, we provided a user-friendly web server (https://tarkg.ddtmlab.org) that enables users to perform knowledge retrieval and relation inference using TarKG.

**Availability and implementation:**

TarKG is accessible at https://tarkg.ddtmlab.org.

## 1 Introduction

Innovative drug discovery is an expensive and risky endeavor, requiring substantial investments of manpower, financial resources, and time. Typically, it takes approximately 12–15 years and $2.8 billion from target identification to the market approval of a new drug (DiMasi 2020, Singh *et al.* 2023). Although around 1700 drugs were approved by FDA since 1906 (Bravo *et al.* 2022, Baedeker *et al.* 2024), a significant proportion of diseases remain inadequately treated, indicating substantial unmet clinical needs for innovative drug discovery. However, the failure rate of new drug clinical trials is currently very high. Insufficient clinical efficacy contributed significantly to the high failure rates of Phase 2 and 3 trials (Sun *et al.* 2022), which is mainly stemmed from inappropriate target selection. Up to 2022, the total number of successfully validated drug targets was <500 (Zhou *et al.* 2022), so target discovery remains a crucial and challenging task to innovative drug discovery. It requires more complex analyses from various perspectives, such as disease biology and chemical intervention, compared with subsequent lead discovery and optimization phases.

In recent years, the development of artificial intelligence (AI) technologies and the accumulation of vast biomedical data have provided important foundations for target discovery as well as other tasks. A prime example is the success of AlphaFold2 (Jumper *et al.* 2021), which provides actionable structural information for protein targets, facilitating research on target biology. For complex biomedical data, knowledge graphs (KGs) are an extremely attractive tool because they offer unique advantages including structural data storage, data integration/association, and knowledge inference. Google pioneered the introduction of the KG concept to enhance web search experiences (Singhal 2012). In 2017, Himmelstein *et al.* created the first comprehensive KG called Hetionet in the biomedical field for drug repurposing (Himmelstein *et al.* 2017). Afterward, various biomedical KGs emerged one after another, such as OpenBioLink (Breit *et al.* 2020), PharmKG (Zheng *et al.* 2020a,b), and PrimeKG (Chandak *et al.* 2023), each typically with different focuses and unique data characteristics. These KGs are being utilized in different scenarios, including drug repurposing, drug–target interactions, among others. However, there is still a lack of KGs specifically focused on target discovery.

Target discovery generally involves comprehensive analysis of data and information from multiple different aspects, with a particular focus on diseases, targets, and intervention molecules, as well as their intricate associations. In comparison, the reported KGs have not provided a sufficiently comprehensive understanding of diseases; e.g. they exhibit individual biases or fail to consider hierarchical relationships among diseases. The existing KGs also do not fully capture and explore the information regarding targets and intervention molecules, especially their relationships with each other, and with diseases. Moreover, Traditional Chinese Medicine (TCM) information has hardly been considered in KGs, which emphasizes the holistic nature of diseases and the harmony of active components, making it a valuable resource for target discovery.

Herein, we describe TarKG (Supplementary Fig. S1), a unified, comprehensive, and large-scale biomedical KG tailored for target discovery. We defined three core entity types closely related to target discovery, including Disease, Gene, and Compound, alongside 12 other associated entity types. We amalgamated and aligned data revolving around these three entity types from seven mainstream biomedical KGs. We further augmented and refreshed information on the core entity types and their associations by leveraging data mining techniques on public resources. In addition, we incorporated four TCM-focused entity types, including Prescription, Chinese materia medica (CMM), Syndrome, and Symptom, along with their associations. TarKG comprises 1 143 313 entities and 32 806 467 triplets cross 15 entity types and 171 relationship pairs. We next assessed the ability of TarKG in knowledge completion, including disease–target link prediction, by using Deep Graph Library Knowledge Embedding (DGL-KE) tools. By using the trained KGE models, we predicted the potential protein targets for Alzheimer’s disease (AD), and explored the unknown diseases potentially associated with the metallo-deubiquitinase CSN5. Furthermore, we offered a user-friendly web server (https://tarkg.ddtmlab.org) to facilitate researchers’ use of TarKG.

## 2 Materials and methods

### 2.1 Data collection, processing, and alignment

TarKG contains three core entity types (Disease, Gene, and Compound), eight associated common entity types (Pathway, Anatomy, Side Effect, Symptom, Phenotype, Biological Process, Molecular Function, and Cellular Component), four TCM entity types (TCM Prescription, TCM CMM, TCM Syndrome, and TCM Symptom), and their relationships represented by triplets. We first integrated and cleaned the data in the existing seven biological KGs, including Hetionet (Himmelstein *et al.* 2017), OpenBioLink (Breit *et al.* 2020), PrimeKG (Chandak *et al.* 2023), PharmKG (Zheng *et al.* 2020a,b), DRKG (Ioannidis *et al.* 2020), MSI (Ruiz *et al.* 2021), and BioKG (Walsh *et al.* 2020). To further enrich the knowledge base and ensure ongoing updates, we incorporated nine mainstream public databases focusing on three core entities, including Disease Ontology (Baron *et al.* 2024), MONDO Ontology (Vasilevsky *et al.* 2022), MESH (Lipscomb 2000), Gene Ontology (Ashburner *et al.* 2000), DrugBank (Knox *et al.* 2024), ChEMBL (Zdrazil *et al.* 2024), INTEDE (Yin *et al.* 2021), E3Atlas (Liu *et al.* 2023a,b), and PubTator3 (Wei *et al.* 2024). We also mined the knowledge related to TCM entities based on eleven important TCM databases, including SoFDA (Zhang *et al.* 2022), TCMIO (Liu *et al.* 2020), ITCM (Tian *et al.* 2023), SymMap (Wu *et al.* 2019), and HERB (Fang *et al.* 2021), among others (Mangal *et al.* 2013, Yan *et al.* 2022, Kong *et al.* 2024, Liu *et al.* 2023a,b, Song *et al.* 2023, Yang *et al.* 2023), and then established relationships with other entities to enhance the comprehensiveness of TarKG. The data sources for these entity types are shown in Supplementary Tables S1 and S2.

#### 2.1.1 Data collection and alignment of entity

We used different methods to align entities for different entity types. In addition, we also enrich node information including text description and structure sequence for three types of core entities: Disease, Gene, and Compound (see details in Supplementary Methods). Finally, we established unified TarKG IDs for all entity types, while retaining their original IDs and sources in a customized mapping table.

#### 2.1.2 Relationship enrichment and triple merging

Triplets are the basic units of a KG, typically represented as <***head entity, relation, tail entity***>, and they play a central role in shaping the KG. All the triplets between the 11 entity types that exist in reported biomedical KGs were initially included. The relationships (triplets) between the three core entity types were then mined and expanded. To gain a more comprehensive understanding of the hierarchical relationship (i.e. is-a) between Disease entities, we analyzed and compiled data from Disease Ontology, MONDO Ontology, and MESH. We incorporated relationships involving Disease, Gene, and Compound entities from Pubtator3 released in 2023 (Wei *et al.* 2024), a resource leveraging AI to extract over a billion entities and relationships from millions of biomedical publications. Given metalloenzymes, drug metabolic enzymes, and E3 ligases are gradually becoming important fields for drug discovery, we expanded their relationships with Disease, Gene, and Compound entities into TarKG by mining MeDBA (Yu *et al.* 2023), INTEDE (Yin *et al.* 2021), and E3Atlas (Liu *et al.* 2023a,b), respectively.

TCM entities were initially linked to other entities within TarKG through the relationships “TCM CMM-Compound” and “TCM Symptom-Symptom.” By combining these with existing relationships, we expanded the connections of TCM entities to other core entities: “TCM CMM-Gene,” “TCM CMM-Disease,” “TCM Prescription-Disease,” “TCM Symptom-Disease,” and “TCM Syndrome-Disease.”

Given the differences in relationship descriptions across various KGs or databases, standardizing relations becomes crucial for deduplicating triples. For the relationships found in existing KGs, we manually standardized them based on their semantics. For instance, relationships such as “CbG” (from Hetionet), “DRUG_BINDING_GENE” (from OpenBioLink), “B” (from PharmKG), and “GNBR::B::Compound: Gene” (from DRKG), were all unified under the term “binds” for Gene and Compound entities. The relationships mined from other resources were uniformly added to TarKG one by one. Notably, we sorted out the directionality of relationships in TarKG. Unreasonable triplets and redundant bidirectional relationships were removed through manual inspection. Triplet deduplication was finalized by substituting entities and relationships where necessary.

#### 2.1.3 Data source tracing and storage

TarKG contained three kinds of data sources, namely mergeKG (from the existing KGs), addKG (from public databases), and tcmKG (from TCM data). Through multiple rounds of entity alignment and relationship integration, each triple and entity in TarKG was tagged with its original data source and index. Duplicate triplets and entities were grouped, and a new index was established using Python, creating a “one-to-many” data traceability mechanism between the current and original data. This enables users to reconstruct the KG based on specific needs (Supplementary Fig. S2). Entity and relation information were separately stored in CSV files in a unified and standardized format. This standardized format and complete traceability ensure ease of updating and utilization for TarKG.

### 2.2 KG embedding (KGE) learning

KGE learning aims to represent entities and relations in a KG as low-dimensional vectors in a continuous vector space, facilitating subsequent tasks such as disease target identification, drug repurposing, and drug–target interaction prediction. Here, we used deep graph library-knowledge embedding (DGL-KE, version 0.1.2) (Zheng *et al.* 2020a,b) for KGE learning, which is an open-source python package containing different KGE algorithms including TransE (Bordes *et al.* 2013), TransR (Lin *et al.* 2015), RESCAL (Nickel *et al.* 2011), DistMult (Yang *et al.* 2014), ComplEx (Trouillon *et al.* 2016), and RotatE (Sun *et al.* 2018) (see Supplementary Table S3). The entire triplets in TarKG were divided into training, validation, and test sets with a 90:5:5 ratio. We used grid search to find the optimal hyperparameters for KGE models (see Supplementary Table S4). Model performance was evaluated using standard ranking metrics: Hits@k (for k ∈ {1, 3, 10}) to assess retrieval success at different ranks, Mean Rank (MR), and Mean Reciprocal Rank (MRR). To evaluate target discovery performance on TarKG, we created a sub-test set focusing on gene–disease triplets from the original test set. Each optimal KGE model underwent individual evaluation.

### 2.3 Prospective analyses of gene–disease link predictions

To further evaluate the predictive capability of TarKG, we used the optimal KGE models (except TransR due to DGL-KE limitations) to predict the potentially unknown-relation between Disease and Gene entities. We used “Gene: Disease::drug targets” as the target relation, and performed two sets of predictions by masking the head entity and tail entity: (i) prediction of potential targets related with Alzheimer’s disease (AD): according to the directionality, Alzheimer’s disease entered as the tail entity (Primary ID: DOID:10652), and the head entity used human Gene entities that are not linked to AD in TarKG; (ii) prediction of COP9 signalosome complex subunit 5 (CSN5)-associated diseases: CSN5 (UniProt ID: Q92905) input as the head entity, and the tail entity used Disease entities that are not linked CSN5 in TarKG. DGL-KE was used to score and rank the entities for prediction within the Gene/Disease Library. The resulting top-ranked entities from the optimal RESCAL model were analyzed through literature analysis.

### 2.4 Web server implementation

To facilitate researchers to use TarKG data, we developed the user-friendly TarKG web server (https://tarkg.ddtmlab.org) that offers entity/path querying, relationship prediction, and graph visualization. We used Neo4j database (version 5.16.0) to store and operate TarKG data, leveraging its superior capabilities to capture the internal relationships among biomedical entities. The web backend uses the Flask web framework (version 3.0.2) to connect to Neo4j Community 5.16 and MySQL Community 8.0, which store TarKG’s current data and original records respectively. The path query request from user on the web page is converted into a Cypher statement supported by Neo4j on the back end. Similarly, DGL-KE is used on the backend to process the link prediction request initiated by the user. The web front-end is built based on the Vue.js (version 3.4.21) framework, and data exchange between the front end and back end is facilitated through API services. Apache ECharts (version 5.5.0) is used to implement graph visualization.

## 3 Results

### 3.1 The profile of TarKG

**Figure 1.**
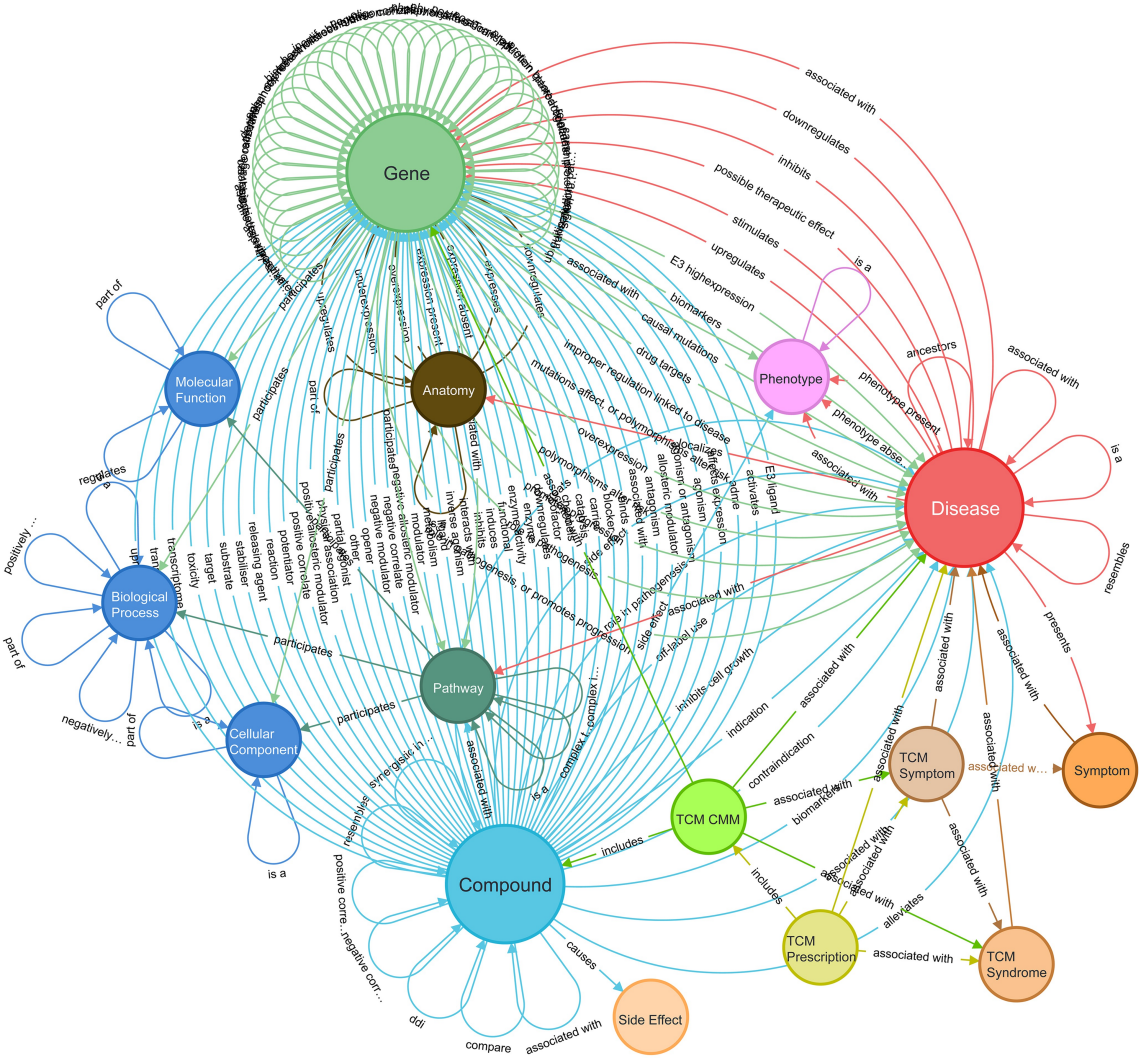
The schema of TarKG. The circles denote entity types, and the links denote relations between entity type pairs. The primary relations revolving around the three core entity types: disease, gene, and compound, can be observed. Detailed relation pairs are given in Supplementary Table S5.

We constructed a holistic KG tailored for target discovery, termed TarKG. Revolving around the three core entity types (Diseases, Gene, and Compound), we integrated data from seven reported biomedical KGs and subsequently expanded and enriched the core entity and relation data from nine public databases (Supplementary Table S2). Further, we uniquely incorporated manually curated Traditional Chinese Medicine (TCM) data, which contain a wealth of valuable information for drug target discovery. Through iterative data integration, alignment, and manual inspection, we obtained a version of TarKG containing 15 entity types and 43 entity pairs (Fig. 1).

Given the diverse data sources used to construct TarKG, particularly the integration of seven existing KGs with varying biological application focuses, establishing a robust data source tracing mechanism is crucial. Each entity and relationship within TarKG retain the original source information, which allows users to not only verify the correctness of knowledge by tracing data back to its origin but also freely reconstruct data from different sources tailored to the specific needs (as illustrated in Supplementary Fig. S2). Notably, in addition to KG sources, users can also rebuild the KG based on the database to which the data belongs.

**Figure 2.**
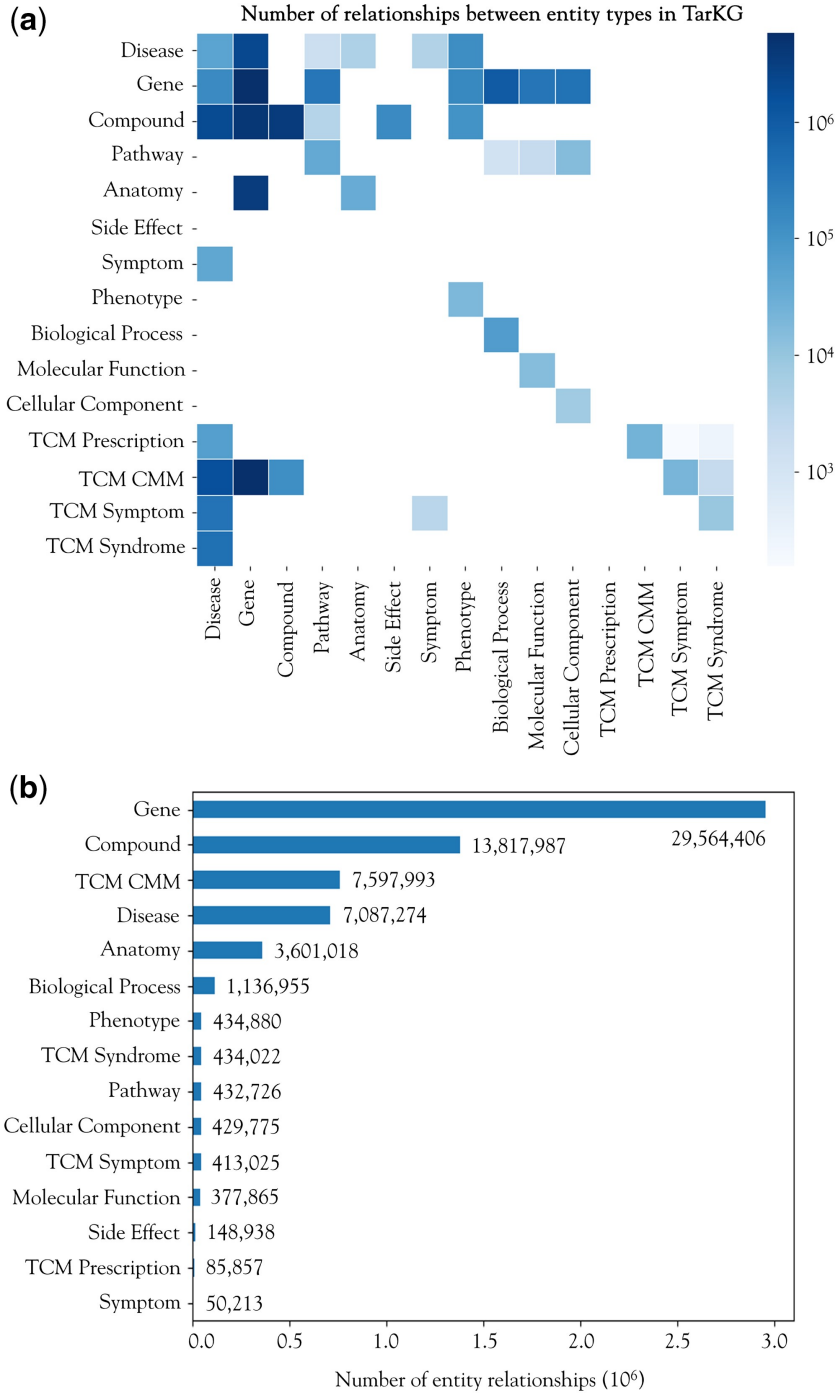
Distribution of entities and relations in TarKG. (a) The heatmap of the number of relations between entity pairs. (b) The number of relations involving the corresponding entity type.

TarKG currently comprises 1 143 313 entities and 32 806 467 triplets, outnumbering almost all reported biomedical KGs (Supplementary Table S6). The million entities are categorized into 15 distinct types, including 31 724 (2.77%) Disease entities, 143 156 (12.52%) Gene entities, and 851 314 (74.46%) Compound entities (Supplementary Table S1). Among the 32 806 467 relationships containing 43 entity pairs, the top 3 pairs constituting nearly half of all triples are: TCM CMM-Gene (17.72%), Gene–Gene (17.35%), and Compound–Gene (12.92%) pairs (Fig. 2a and Supplementary Table S7). Analysis of the number of relationships each entity participates in revealed that Gene (45.06%), Compound (21.06%), TCM CMM (11.58%), and Disease (10.80%) are the most interconnected entities (Fig. 2b and Supplementary Table S8). In addition, the Compound–Gene, Gene–Gene, and Gene–Disease pairs exhibit the greatest diversity in relationship types, with 45, 34, and 11, respectively. These features reflect that TarKG is a comprehensive biomedical KG centered around Compound, Disease, and Gene, and meanwhile, enriched with distinctive features of Traditional Chinese Medicine.

### 3.2 The characteristics of TarKG for target discovery

Compared with reported biomedical KGs, TarKG possesses the following characteristics specifically tailored for target discovery. The first major advantage of TarKG lies in its multi-dimensional consideration of the three core entity types and their diverse relations closely related to target discovery, along with specialized data integration and mining. Given the abundance, diversity, and lack of uniformity in Disease entities, we built a detailed Disease ID mapping pool covering Disease entity terms from multiple disease databases such as Disease Ontology, MONDO Ontology, MESH, OMIM, CTD, and UMLS. This addresses the issue encountered in previous KGs, where only one type of Disease ID identifier was included. TarKG features twice the number of diseases (31 724 entities) compared to PrimeKG, a previously reported KG known for its extensive collection of Disease entities. There are 7 087 274 relationships involving Disease entity type, reflecting a wealth of disease information within TarKG.

**Figure 3.**
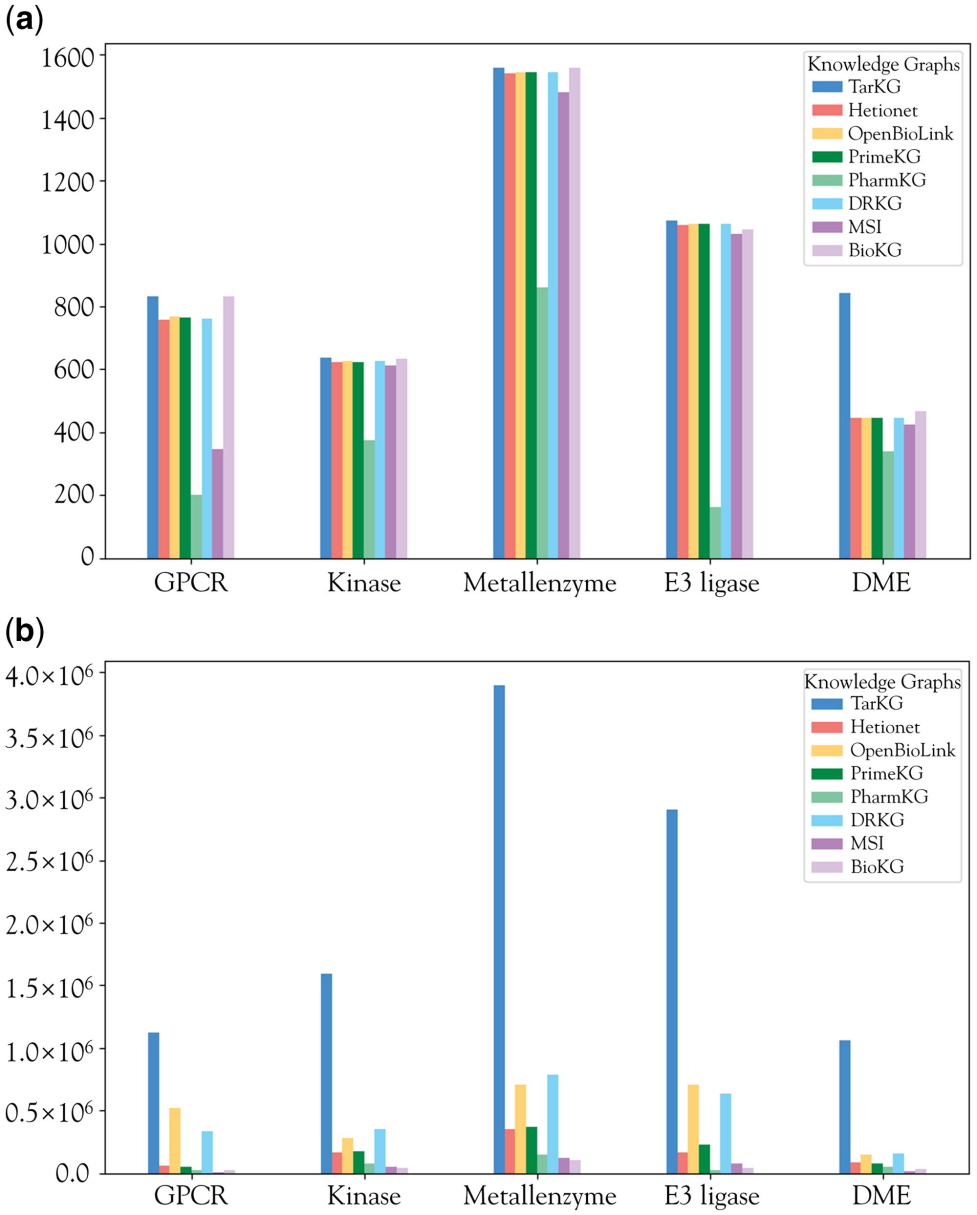
Comparison of main target groups in different KGs. (a) The number of target entities within GPCRs, kinases, metalloenzymes, E3 ligases, and DMEs; (b) the number of target relations within the five target groups.

For Gene entities, we mainly consider human proteins or other protein targets related to human diseases. We have specifically expanded the information for G protein-coupled receptors (GPCRs), kinases, metalloenzymes, E3 ligases, and drug-metabolizing enzymes (DMEs), as they are main groups of potential drug targets. While the number of entities for these target groups, except for DMEs, in TarKG is comparable to other KGs (Fig. 3a), TarKG has a remarkable increase (2- to 10-fold) in the number of relationships associated with these targets (Fig. 3b), reflecting more enriched relational information, which facilitates inference of new relationships.

Compounds are the largest number of entities in TarKG, accounting for as much as 74.46% of the total. To better meet the needs for pharmaceutical researchers and others, we labeled “drugs” that are either on the market or being evaluated in clinical trials within the Compound entities, which is uncommon in reported KGs.

Another major superiority of TarKG is the inclusion of TCM data, which is rarely considered but highly valuable. Given their diverse active chemical components and multi-target synergistic effects, TCM data are a valuable source for innovative drug discovery. TarKG integrated TCM data into existing biomedical KGs, aiming to bridge the gap between isolated TCM entities and the modern biomedical relationship network. Despite constituting a mere 0.74% of all entities in TarKG, TCM entities are remarkably involved in 13% of all recorded relationships. Notably, TCM CMM entities account for 11.58% of all relationships, ranking third in terms of relationship richness. Its prominence extends to its interactions with the three core entities: Genes, Diseases, and Compounds. The inclusion of such data in TarKG allows for a deeper exploration of disease-gene relationships, potentially unearthing novel insights and providing new perspectives in drug discovery and therapeutic target identification.

We provided additional node information for Diseases, Genes, and Compounds. Specifically, disease features are represented using textual descriptions (e.g. names, definitions, and synonyms), while compound and gene features incorporate both structural/sequential data and textual descriptions. In short, given these distinctive features, TarKG has a great potential in target discovery and other tasks.

### 3.3 TarKG-based KG completion

We next examined the ability of TarKG in KG completion. Six KGE algorithms, including TransE, TransR, RESCAL, DistMult, ComplEx, and RotatE, are used for graph learning by computing machine-readable embedding vectors of entities and relationships in TarKG. Table 1 presents the performance of each model on the independent test set. All six KGE models showed good performance in relation prediction, with Hits@3 scores exceeding 0.9, except for DistMult. Particularly, TransR and RESCAL exhibited exceptional performance, with both achieving Hits@1 scores of 0.87. Compared with previous KGs such as PharmKG and Hetionet, all these KGE models perform better on TarKG (Zheng *et al.* 2020a,b). This improved performance is at least partially attributed to TarKG’s greater number of many-to-many relationships; in TarKG, a given head entity and relationship are associated with more potential tail entities, increasing the likelihood of correct predictions.

**Table 1.** Performance of KGE models in relation prediction on the test set.

Metric^a^	TransE (L2)	TransR	RESCAL	DistMult	ComplEx	RotatE
MRR	0.83	0.91	0.91	0.83	0.84	0.85
MR	7.09	8.01	7.56	8.44	7.31	11.22
Hits@1	0.75	0.87	0.87	0.75	0.77	0.77
Hits@3	0.90	0.94	0.95	0.89	0.90	0.90
Hits@10	0.97	0.97	0.97	0.96	0.96	0.96

a MR: Mean Rank; MRR: Mean Reciprocal Ranking; Hits@k (*k* = 1, 3, or 10): average proportion of triples with rank <*k* in link prediction. The lower the MR value, the higher the MRR and Hits@k values, indicating that the model prediction performance is better.

To specifically evaluate gene–disease relationship prediction, we constructed a sub-test set containing 118 083 gene–disease links (accounting for 7% of the total relationships in the test set), which features diverse non-one-to-one relationships. We observed that TransR and RESCAL manifested superior performance on the gene–disease subset compared to whole test set, with MRR and Hits@1 scores excelling 0.9 and 0.87, respectively, while other models showed relatively lower performance on the subset (Table 2). TransR improves flexibility and expressiveness through mapping of relationship-specific spaces, and RESCAL captures high-order interactions through high-dimensional representation and tensor decomposition. These features make them more advantageous in dealing with gene–disease subset with fewer but more complex relationships. In contrast, other models like TransE and DistMult, with simpler representations, may struggle with such complex relationships, potentially leading to decreased prediction accuracy. The results partly reflected the challenge of inferring gene–disease relationships given their complex internal relationships, and more importantly, highlighted the necessity of developing KGE models specifically suited for gene–disease relationship prediction.

**Table 2.** Performance of KGE models in relation predictions on gene–disease subset.

Metric	TransE (L2)	TransR	RESCAL	DistMult	ComplEx	RotatE
MRR	0.75	0.93	0.92	0.71	0.71	0.75
MR	3.64	1.44	1.46	6.82	4.45	4.26
Hits@1	0.63	0.88	0.87	0.58	0.58	0.64
Hits@3	0.83	0.97	0.97	0.80	0.81	0.83
Hits@10	0.95	1.00	1.00	0.93	0.94	0.95

### 3.4 Case studies

DGL-KE supports link prediction using various KG embedding (KGE) models, including TransE, RESCAL, DistMult, ComplEx, and RotatE. We used these KGE models to predict the potential protein targets for AD and to identify potentially related diseases for the metallo-deubiquitinase CSN5 in TarKG.

***Potential targets for AD*.** Using different KGE models, we observed distinctly different prediction results. Only a few targets were consistently ranked within the top 10 by multiple KGE models, such as KCHN2 (UniProt ID: Q12809) and BAD (UniProt ID: Q92934) (Supplementary Table S9). Given RESCAL’s superior performance on the gene–disease sub-test set (Table 2), we conducted a detailed literature analysis for the top 10 predictions. We found that nine of the 10 predicted targets (90% success rate) are supported by existing literature (Supplementary Table S10). For example, the top1-ranked predicted target for AD is Bcl2-associated agonist of cell death (BAD), a member of the Bcl-2 family. BAD is associated with cellular apoptosis, a process implicated in AD and other neurodegenerative diseases (Obulesu and Lakshmi 2014), and the observed upregulation of BAD protein expression in AD brains (Kitamura *et al.* 1998) suggest its potential as a therapeutic target. We also utilized the Neo4j graph database to explore the existing paths between BAD and AD within TarKG (Supplementary Fig. S3). Likewise, xanthine dehydrogenase (XDH) ranked second is a hydroxylase involved in purine oxidation metabolism. This metabolic pathway is known to contribute to the generation of reactive oxygen species (ROS), another hallmark of AD (Pathak *et al.* 2019). Furthermore, lipid dysregulation was identified recently as a potential contributing factor to AD (Yin 2023). Diacylglycerol O-acyltransferase 1 (DGAT1), the third-ranked predicted target, plays a critical role in regulating the biosynthesis of lipid droplets (LDs), which are intimately involved in lipid metabolism (Yang *et al.* 2022).

***Potentially related diseases of CSN5***. Similarly, the five KGE models yielded varying results in predicting CSN5-associated diseases. Only myelogenous leukemia (Primary ID: MESH:D007951) consistently appeared in the top 10 disease list predicted by different models (Supplementary Table S11). We conducted further literature analysis on the top 10 diseases predicted by RESCAL (Supplementary Table S12). Out of the ten predicted diseases, seven diseases have documented associations supported by literatures. For example, the top-3 predicted disease was progressive transformation of germinal centers (Primary ID: MONDO:0043346), which aligns with prior findings that Jun activation domain-binding protein 1 (Jab1, another name of CSN5) is crucial for the expression of Bcl6, a transcription repressor required for germinal center formation (Sitte *et al.* 2012). Likewise, Haemophilus Infectious Disease (Primary ID: MONDO:0006926) and Precursor T-Cell Lymphoblastic Leukemia-Lymphoma (Primary ID: MESH:D054218), ranked fourth and fifth respectively, have both been shown to be associated with the ubiquitination pathway (Li and Zhong 2018, Fhu and Ali 2021). Given the crucial role of CSN5, a metallo-deubiquitinase, in the ubiquitination system by regulating activity of cullin-RING E3 ubiquitin ligases, it is likely that these two diseases are associated with CSN5. Furthermore, previous studies suggested that the Jab1/CSN5 signaling pathway has a close association with the 5-HT6 receptor (5-HT6R) (Chaumont-Dubel *et al.* 2020) that may play a crucial role in epileptogenesis and cognitive impairment (Liu *et al.* 2019, Chaumont-Dubel *et al.* 2020), which partly support the top 6, 9, and 10 prediction results (Supplementary Table S12). These results reveal the potential of TarKG in identifying new possible Disease-Gene relations.

### 3.5 The web server of TarKG

**Figure 4.**
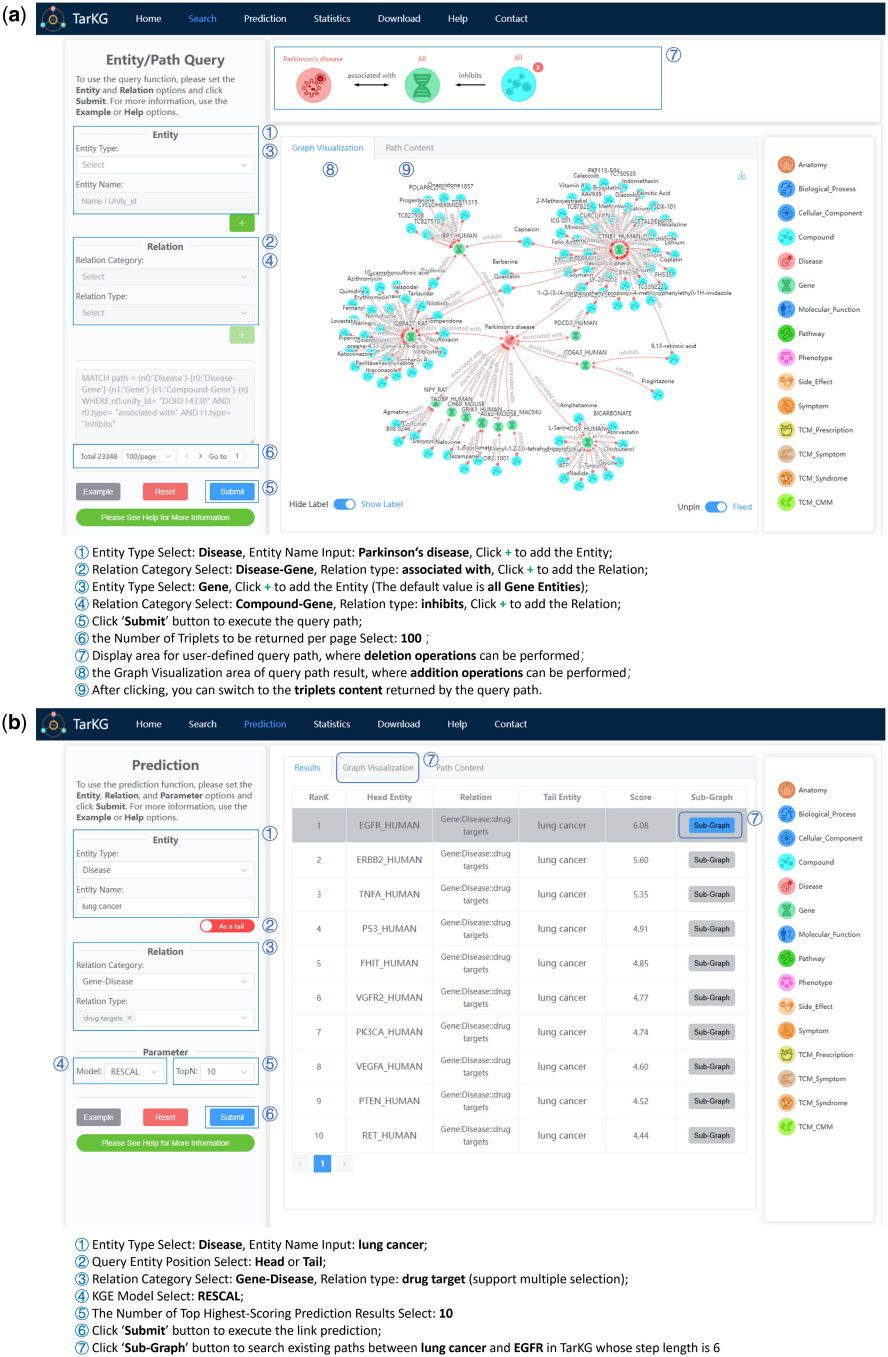
User interface of the TarKG web server. (a) Showing a usage process for knowledge retrieval: identification of inhibitors for PD-associated genes through a two-hop path query. (b) Showing a usage process for relation inference: prediction of potential targets for lung cancer.

To facilitate user access and download of TarKG data, we established a user-friendly web server (https://tarkg.ddtmlab.org). This server enables users to construct custom queries through intuitive clicking operations. It contains two functional modules: knowledge retrieval and relation inference. Figure 4a shows knowledge retrieval using a path query example, i.e. the identification of Parkinson’s disease (PD) associated genes and their inhibitors through a two-hop path: Disease (PD) &cenveo_unknown_entity_wingdings_F0DF; associated with → Gene &cenveo_unknown_entity_wingdings_F0DF; inhibits–Compound. For this purpose, we can firstly query for all Gene entities associated with PD, and then identify all Compound entities that exhibit an inhibitory effect on these Genes. By limiting the returned/visualized results per page to the top 100, we can obtain the results shown in Fig. 4a. For example, pioglitazone has been shown to inhibit the PD-associated gene COL6A3 (Jin *et al.* 2021), indicating its potential relevance to PD. This prediction is supported by a previous meta- analysis indicating that administering pioglitazone is associated with reduce risk of PD in diabetes patients (Chen *et al.* 2022).

Figure 4b illustrates the workflow of relation inference, taking the prediction of potential targets for lung cancer as an example. User can initiate the query by selecting the disease “lung cancer” as the tail entity, and set the relationship as “Gene: Disease::drug target.” By default, the head entity pool is populated with all gene entities present within TarKG. By selecting the RESCAL model for KGE analysis and limiting the results to the top 10, user can see the top 10 predicted targets for lung cancer on the page. For instance, epidermal growth factor receptor (EGFR), ranked as the top 1 in the prediction results, has been found to be associated with certain lung cancers (Bethune *et al.* 2010). Notably, for each predicted result, user can obtain the existing paths within TarKG (up to six steps in length) by clicking the “Sub-Graph” button. The web server also offers statistical data of TarKG and allows users to access detailed entity and relation information stored in .csv files for both TarKG and original data.

## 4 Discussions

TarKG was created by integrating biomedical KGs, public data resources and TCM data. It forms a large-scale and high-quality biomedical KG with a focus on the three core entity types and their relations. The currently largest number of Disease entities and relations in TarKG holds tremendous potential for addressing disease target discovery and clarifying disease mechanisms. Expanded data for the important target groups, such as metalloenzymes, E3 ligases, and drug-metabolizing enzymes, have been incorporated into TarKG, thereby enhancing the coverage of these target datasets. Compounds, as crucial bridges between targets and diseases, have also been subjected to focused data mining and labeled at different stages, which will facilitate drug target discovery and other purposes. In addition, TarKG uniquely incorporates TCM data, which are rich and valuable sources for drug synergistic effects and drug-diseases associations.

Target discovery often demands a comprehensive understanding of disease mechanisms, which involves exploring various biomedical entities and their complex relationships. This requires rich biological network information as well as specialized knowledge of the relevant core entities. TarKG, with its extensive data coverage and focus on disease-related entities/relationships, serves as a unique and valuable resource for meeting these requirements. Moreover, the large-scale entity and relation data in TarKG can be utilized for various other purposes, such as drug repurposing, protein–ligand interactions, and TCM efficacy understanding.

The preliminary results of KGE training on TarKG revealed its strong capability for KG completion and target prediction (either from disease to targets or from target to diseases), reflecting the effective construction and data quality of TarKG. In addition to data quality and comprehensiveness, KG reasoning methods are also crucial factors affecting their accuracy in link prediction. Among the six KGE models, TransR and RESCAL manifested superior performance on disease-gene link prediction, possibly attributed to their capability in handling relationship-specific spaces. Recently, researchers have started developing more advanced KG reasoning models by incorporating domain-specific knowledge of biological entities into foundation models. For example, DREAMwalk (Bang *et al.* 2023) used a semantic information-guided random walk to generate node embeddings for drugs and diseases, facilitating drug repositioning. KGCNH (Du *et al.* 2024) utilized a graph convolutional network based on heuristic search to tackle drug repurposing. Since most of these models are primarily focused on tasks such as drug repositioning and drug–target interaction prediction, there remains considerable potential for developing reasoning models specifically designed for target discovery.

During the construction of TarKG, we observed that entity and relationship alignment methods are still not perfect, leading to the loss of valuable data in some cases, which is a key issue that needs to be addressed in the future. Furthermore, the challenge of fully deduplicating entities persists due to incomplete cross-mapping of entity IDs across different databases. For example, TarKG still contains a small number of disease entities that share the same name but have different IDs. To address this issue and enhance data reliability, we have established a comprehensive data traceability mechanism that allows users to verify the accuracy of the knowledge by accessing the source information of each data entry. In addition, this mechanism enables users to reconstruct the KG with a focus on different data sources, facilitating the creation of benchmark datasets for specific tasks and supporting the advancement of KG reasoning methods. We believe that these rigorous data, combined with stronger KG reasoning methods, will be a powerful resource for target discovery and other applications.

## Supplementary Material

btae598_Supplementary_Data

## Data Availability

All data used in this paper are downloaded from https://tarkg.ddtmlab.org/download
