# Supplementary material for: TarKG: a comprehensive biomedical knowledge graph for target discovery
Source: Bioinformatics. 2024 Oct 11;40(10):btae598. doi: 10.1093/bioinformatics/btae598 (PMC11513019; doi:10.1093/bioinformatics/btae598)
Supplement: btae598_Supplementary_Data [file btae598_supplementary_data.zip › Supplementary Materials for Online.docx]

*Supplementary Data*

**TarKG: A Comprehensive Biomedical Knowledge Graph for Target Discovery**

Cong Zhou^1,†^, Chui-Pu Cai^2,†^, Xiao-Tian Huang^1^, Song Wu^1^, Jun-Lin Yu^1^, Jing-Wei Wu^1^, Jian-Song Fang^3,*^, and Guo-Bo Li^1,*^

^1^Key Laboratory of Drug-Targeting and Drug Delivery System of the Education Ministry and Sichuan Province, Department of Medicinal Chemistry, West China School of Pharmacy, Sichuan University, Chengdu 610041, China

^2^Division of Data Intelligence, Department of Computer Science, Shantou University, Shantou 515063, China

^3^Science and Technology Innovation Center, Guangzhou University of Chinese Medicine, Guangzhou 510405, China

*To whom correspondence should be addressed: [liguobo@scu.edu.cn](mailto:liguobo@scu.edu.cn) (G.-B.L.) or [fangjs@gzucm.edu.cn](mailto:fangjs@gzucm.edu.cn) (J.-S.F.)

^†^C.Z. and C.-P. C. are co-first authors

**Contents**

**1. Supplementary Methods** S3

1.1. Data collection and alignment methods for each entity typeS3

1.2. Node information enrichment of three core entity typesS6

**2. Supplementary Tables** S7

**Table S1**. The number and sources of entity types included in TarKG.S7

**Table S2**. The introduction of data sources for constructing TarKG.S8

**Table S3**. Brief descriptions of the six KGE algorithms.S13

**Table S4**. The optimal hyperparameters of each KGE model on TarKG.S14

**Table S5**. The type and number of relationships contained by each entity pair in the TarKG.S15

**Table S6**. Comparation of TarKG with other biomedical knowledge graphs.S22

**Table S7**. The number of relations of different entity pairs in TarKG.S23

**Table S8**. The number and proportion of relationships involving entities in TarKG.S25

**Table S9**. List of the top 10 potential protein targets for AD with different KGE models.S26

**Table S10**. Literature analysis of the top 10 potential protein targets for AD predicted with RESCAL.S26

**Table S11**. List of the top 10 of potentially related diseases for CSN5 with five KGE models.S28

**Table S12**. Literature analysis of the top 10 potentially related diseases for CSN5 with RESCAL.S28

**3. Supplementary Figures** S31

**Figure S1**. Pipeline for TarKG construction, web server and applications. (a) The main data components and sources of TarKG. (b) Data process of TarKG construction. (c) Web server and applications of TarKG.S31

**Figure S2**. The mechanism sample graph of data tracing and knowledge graph reconstruction in TarKG.S32

**Figure S3**. Graphical illustration of partial existing paths between BAD and AD in TarKG, with a path length of 6.S32

**Reference** S33

**1. Supplementary Methods**

**1.1. Data collection and alignment methods for each entity type**

***Disease***. Accurate and comprehensive disease information are crucial for target discovery. The hierarchical relationships among diseases and disease-related associations were thoroughly considered here. Since the data sources of diseases and associations are diverse and non-uniform, we established an ID mapping pool to align Disease entities from different databases. Disease entity sources mainly include DO (Baron et al. 2023), MONDO (Vasilevsky et al. 2022), MeSH (Lipscomb 2000), OMIM (Amberger et al. 2018), CTD (Davis et al. 2022), and UMLS (Bodenreider 2004). We initialized the mapping pool using DO IDs. Subsequently, missing entity IDs from other databases were filled in according to the specified sequence (*i.e.*, DO, MONDO, MESH, OMIM, CTD, and UMLS). UMLS, ICD9, and ICD10 were used as bridges for unifying Disease entities across multiple databases. Notably, we included genetic disorders from OMIM as a Disease entity type, which were treated as an independent entity type in other knowledge graphs. Lastly, the original IDs of the Disease entities were replaced with the primary keys from the disease ID matching table. To ensure data quality, rigorous manual checks and method optimization were performed iteratively.

***Gene***. Genes were regarded as the core entity type due to their broad associations with diseases and their extensive involvement in gene-gene networks. Gene information was collected from two public sources: Entrez Gene (Maglott et al. 2010) and UniProt (Consortium 2022). All genes were unified by UniProt IDs, with the aim to mainly focus on protein targets. To avoid one-to-many correspondences, we only retain the first UniProt ID when converting from gene IDs, prioritizing Swiss-Protein entries. Approximately 10% of gene IDs were dropped due to their un-matched relations with UniProt IDs. Furthermore, to ensure comprehensive knowledge coverage, we also included non-human proteins in TarKG, as long as they are connected by relationships within the knowledge graph.

***Compound***. As key bridges between diseases and targets, intervention molecules were treated as the core entity type. The intervention molecules were collected from multiple databases, including DrugBank (Knox et al. 2023), ChEMBL (Zdrazil et al. 2023), PubChem (Kim et al. 2022), ChEBI (Hastings et al. 2015), MeSH, and others. Among them, drug molecules and drug candidates were mainly collected from DrugBank, PubChem, and CHEBI, and experimental compounds were mainly from ChEMBL (version 33). The chemical structures (SMILES or InChI) for all compounds were downloaded from the respective databases or PubChem. All chemical structures were converted to standardized InChIs by using OpenBabel (version 2.4.1) (O'Boyle et al. 2011). The Compound entities lacking of chemical structures were removed. Duplicated entities were removed according to the standardized InChIs.

***Pathway***. The Pathway entities are closely related with genes and diseases. We collected Pathway entities from Pathway Commons (Rodchenkov et al. 2019), WikiPathways (Agrawal et al. 2023), PathBank (Wishart et al. 2023), KEGG (Kanehisa et al. 2022), and REACTOME (Milacic et al. 2023). Since none of these databases provide ID mappings of pathways between each other, we aligned them based on pathway names. However, we encountered inconsistencies in pathways with the same name across different databases. To address this, we first removed non-human pathways and then deduplicated the remaining pathways using their original database IDs, ensuring the removal of redundant entities.

***Gene Ontology***. A glossary for describing gene functions, dividing into three branches (entities): Cellular Component, Biological Process, and Molecular Function, were included in TarKG. All the original IDs and branch types were collected from the last version of Gene Ontology (Ashburner et al. 2000).

***Anatomy***. Anatomy plays a key role in understanding disease occurrence and targeting therapies. Currently, four knowledge graphs incorporated Anatomy entities. While a small portion originates from the Cell Ontology (Diehl et al. 2016), the primary source is Uberon (Haendel et al. 2014), a comprehensive multi-species anatomy ontology encompassing human body parts, organs, and tissues. To align Anatomy entities, TarKG retains the original identifiers from Uberon and Cell Ontology.

***Side Effect***. The Side Effect entity is closely related with drug targets. The side effect information was collected from the Side Effect Resource (SIDER) (Kuhn et al. 2015); the identifiers of Side Effect entities were unified into UMLS.

***Symptom***. Symptom is the explicit manifestation of disease in the human body, serving as an important bridge linking Disease entities and TCM Symptom entities in TarKG. Only Hetionet and DRKG provides Symptom entities whose terminology source is MESH. Therefore, we employed the Disease ID mapping pool to unify identifier of Symptom entities.

***Phenotype***. The Phenotype entities were collected from the Human Phenotype Ontology (HPO) (Gargano et al. 2023), providing a standardized lexicon encompassing the phenotypic abnormalities observed in human diseases. HPO IDs were used as the Phenotype entity identifiers in TarKG.

***TCM Prescription***: The TCM prescriptions are formulated combinations of herbs, minerals, and animal products, which are tailored based on a comprehensive diagnosis that considers the individual's overall health, symptoms, constitution, and both external and internal factors influencing their well-being. We primarily collected 2,626 prescriptions from the SoFDA platform (Zhang et al. 2022), the Traditional Chinese Medicine on Immuno-Oncology (TCMIO) database (Liu et al. 2020), and over 100 Chinese medical classic books, which includes the prescriptions listed in the Chinese Pharmacopoeia as well as those of approval marketed Chinese patent medicines. In TarKG, each TCM prescription has been assigned a unique identifier.

***TCM CMM***: A total of 3,211 TCM CMMs were gathered from traditional Chinese medicine (ITCM) (Tian et al. 2023), SymMap (Wu et al. 2018), TCMIO (Liu et al. 2020) and HERB databases (Fang et al. 2020), as well as our previous works (Cai et al. 2021; Huang et al. 2019). Meanwhile, we further collated the detailed information of CMMs from multiple authoritative TCM databases, Chinese Pharmacopoeia, specifications and literatures, including property, flavor, meridian tropism, indication, function, species, toxicity, etc, and established 124,806 pairs of relationship between Chinese medicinal herbs and their corresponding active chemical ingredients (Kong et al. 2023; Liu et al. 2023; Song et al. 2023; Yang et al. 2023; Zhang et al. 2022).

***TCM Syndrome***: TCM syndromes are distinctive symptom groups summarized based on TCM theories and thousands of years of clinical empirical knowledge, considered as the core diagnostic criteria and therapeutic guidance. By retrieving the Chinese Pharmacopoeia (version 2015 and 2020), we obtained 232 TCM syndromes and incorporated into TarKG with unique entity identifiers. Besides, 2,104 pairs of TCM syndrome-TCM CMM associations were supplemented according to the SymMap database (Wu et al. 2018).

***TCM Symptom:*** The knowledge of TCM symptoms and their interrelationships with TCM CMMs and TCM syndromes were primary curated based on SymMap (Wu et al. 2018). To bridge the gap between traditional Chinese diagnoses and modern medical diagnoses, the 2,357 TCM symptoms were linked to modern medical symptoms with MESH, DOID or UMLS ID under the guidance of experts practicing TCM.

**1.2. Node information enrichment of three core entity types**

Specifically, for the three core entity types (Disease, Gene, and Compound), we have gathered additional information reflecting their specific characteristics. The Disease entity comprises disease name, definition, and synonym. The Gene and Compound entities encompass structural features (represented by protein sequences) and the InChI/SMILES strings, respectively. Additionally, the drugs or drug candidates categorized under the Compound entity type were documented with their highest clinical status sourced from DrugBank and ChEMBL.

**2. Supplementary Tables**

**Table S1**. The number and sources of entity types included in TarKG.

| Entity Type | Count | Percent | Data Source |
| --- | --- | --- | --- |
| Compound | 851,314 | 74.46% | Hetionet, OpenBioLink, PrimeKG, PharmKG, DRKG, MSI, BioKG, DrugBank, ChEMBL, Pubtator3, INTEDE, TCMIO, SoFDA, ITCM, SymMap, HERB, ccTCM, DCABM-TCM, TCMSTD, BATMAN-TCM 2.0, NPACT, HIT 2.0 |
| Gene | 143,156 | 12.52% | Hetionet, OpenBioLink, PrimeKG, PharmKG, DRKG, MSI, BioKG, Pubtator3, INTEDE, E3Atlas, TCMIO, ITCM, HERB, NPACT, HIT 2.0 |
| Disease | 31,724 | 2.77% | Hetionet, OpenBioLink, PrimeKG, PharmKG, DRKG, MSI, BioKG, Disease Ontology, MONDO Ontology, MESH, PubTator3, INTEDE, E3Atlas |
| Pathway | 28,162 | 2.46% | Hetionet, OpenBioLink, PrimeKG, DRKG, BioKG |
| Biological Process | 27,538 | 2.41% | Hetionet, OpenBioLink, PrimeKG, DRKG, MSI, BioKG, Gene Ontology |
| Anatomy | 16,031 | 1.40% | Hetionet, OpenBioLink, PrimeKG, DRKG |
| Phenotype | 14,715 | 1.29% | OpenBioLink, PrimeKG |
| Molecular Function | 11,239 | 0.98% | Hetionet, OpenBioLink, PrimeKG, DRKG, MSI, BioKG, Gene Ontology |
| Side Effect | 5,722 | 0.50% | Hetionet, DRKG, BioKG |
| Cellular Component | 4,060 | 0.36% | Hetionet, OpenBioLink, PrimeKG, DRKG, MSI, BioKG, Gene Ontology |
| TCM CMM | 3,211 | 0.28% | TCMIO, SoFDA, ITCM, SymMap, HERB, ccTCM, DCABM-TCM, TCMSTD, BATMAN-TCM 2.0, HIT 2.0 |
| TCM Prescription | 2,626 | 0.23% | TCMIO, SoFDA |
| TCM Symptom | 2,357 | 0.21% | SymMap |
| Symptom | 1,226 | 0.11% | Hetionet, DRKG |
| TCM Syndrome | 232 | 0.02% | SymMap |
| Total | 1,143,313 | 100.00% | - |

**Table S2**. The introduction of data sources for constructing TarKG.

| **Data Sources** | **Description** | **Entity Types^a^** |
| --- | --- | --- |
| **Hetionet v1.0** | The first knowledge graph integrating public biomedical resources to help drug repurposing was Hetionet, which comprises 47,031 nodes (11 types) and 2,250,197 relations (24 types). Data was mined from 29 databases of genes, compounds, diseases, and more, including DrugBank, Disease Ontology, Gene Ontology, SIDER, WikiPathways, Uberon, and so on. Hetionet was created for Project Rephetio, a study to systematically evaluate why drugs work and to predict new therapeutic uses for existing drugs. | Disease, Gene, Compound, Pathway, Anatomy, Side Effect, Symptom, Biological Process, Cellular Component, Molecular Function |
| **PharmKG** | PharmKG is aimed at constructing a high-quality biomedical knowledge graph and designing novel GNN-based KGE method to address various tasks in drug discovery. PharmKG consists of 7,600 entities and ~50,000 relationships of 29 types, and entities is only three types: gene, chemicals, and disease. The data is obtained from seven public databases: OMIM, DrugBank, PharmGKB, Therapeutic Target Database (TTD), SIDER, HumanNet, and GNBR, an interaction network extracted from extensive biomedical literature. Compared to other knowledge graphs, PharmKG incorporates numerical features for all entities. | Disease, Gene, Compound |
| **OpenBioLink** | OpenBioLink serves as the resource framework for assessing different link prediction models on heterogeneous biomedical graph data. A 5 million-level benchmark data set containing 7 node and 30 edge types was created from 17 databases, such as DisGeNET, STRING, and CTD. To better apply various KG reason methods, OpenBioLink benchmark-graph offers both directed and undirected versions, and four confidence level versions (high, medium, low, and all). More importantly, OpenBioLink collected true negative triplets to avoid appearance of false-negative when model performs negative sampling strategy. | Disease, Gene, Compound, Pathway, Anatomy, Phenotype, Gene Ontology |
| **DRKG** | Based on Hetionet, Drug Repurposing Knowledge Graph (DRKG) extracted data from six databases: DrugBank, Hetionet, GNBR, String, IntAct, and DGIdb, along with information gathered from publications particularly related to Covid19. DRKG consists of 97,238 entities across 13 entity types, and 5,874,261 triplets across 107 edge types. DRKG also provides pre-trained embedding vectors for entities and relations using Knowledge Graph Embedding (KGE), as well as molecular feature representations based on graph neural networks. Lastly, the performance of DRKG was evaluated in drug repurposing tasks related to COVID-19. | Disease, Gene, Compound, Pathway, Anatomy, Side Effect, Symptom, Biological Process, Cellular Component, Molecular Function |
| **BioKG** | BioKG integrated 13 open-source databases and undergone a high-quality data cleaning process, which comprises ~105,000 entities and 2 million relationships for biomedical graph relation learning. The content of BioKG primarily includes three categories: links, properties, and metadata. The links section is the core of BioKG, used to construct TarKG, along with partial data from the properties section. | Disease, Gene, Compound, Pathway, Side Effect, Gene Ontology |
| **MSI** | The multiscale interactome (MSI) network consists of four types of entities: Drug, Protein, Disease, and Geno Ontology Biological Function, along with five types of interactions among them. The authors computed diffusion profiles for every node using a power matrix formulation and performed pharmacological tasks such as disease treatment mechanisms analysis and drug repurposing. | Disease, Gene, Compound, Gene Ontology |
| **PrimeKG** | PrimeKG is a precision medicine-oriented multimodal knowledge graph with 129735 nodes of 10 types and 4050249 edges of 30 types. For aiding personalized diagnostic and treatment strategies, PrimeKG provides a larger set of disease terms (22,236) and collects clinical features of diseases and drugs by integrating 20 high-quality resources. And in terms of data format, PrimeKG is the simplest and most user-friendly. | Disease, Gene, Compound, Pathway, Anatomy, Phenotype, Biological Process, Cellular Component, Molecular Function |
| **Disease Ontology** | Disease Ontology (DO) is a standardized ontology including 11,300 common and rare diseases, with cross-mapping of vocabulary for 37,000 disease concepts. | Disease |
| **MeSH** | Medical Subject Headings (MeSH) is a medical vocabulary database established by the National Library of Medicine. MeSH records subject descriptors appearing in PubMed and other NLM databases, and the descriptors are grouped into 16 categories: anatomic terms, diseases, chemicals, and so on. | Disease, Compound |
| **MONDO** | MONDO was created to harmonize disease definitions through integrating approximately 90000 source concepts from 17 disease resources into 22,157 distinct disease concepts | Disease |
| **DrugBank** | DrugBank is a canonical and comprehensive drug database, which records basic drug information and relationships of drug-drug, drug-pathway, and drug-target. | Disease, Gene, Compound, Pathway |
| **ChEMBL** | ChEMBL is a manually curated, large-scale, open database of bioactive molecules, containing a vast amount of activity data for compounds and also collects indications for clinical drugs. | Disease, Gene, Compound |
| **Gene Ontology** | Gene Ontology (GO) is a holistic resource focusing on gene function and gene products, which divides biological domain knowledge into three parts: Molecular Function, Cellular Component and Biological Process. | Cellular Component, Biological Process, Molecular Function |
| **Pubtator3** | Pubtator3 utilized state-of-the-art AI technology to extract entities and relationships from biomedical literature, and keeps updated weekly. Currently, it has mined over 1 billion entities and relationships including diseases, proteins, and chemicals, from 36 million abstracts and 6 million full-text articles. | Disease, Gene, Compound |
| **INTEDE** | Interactome of Drug-Metabolizing Enzymes (INTEDE) was constructed to offer the interactome of 1047 unique drug-metabolizing enzymes (DMEs), mainly including 3 interaction types: microbiome–DME interaction (MICBIO), xenobiotics–DME interaction (XEOTIC) and host protein–DME interaction (HOSPPI). | Disease, Gene, Compound |
| **E3Atlas** | E3Atlas collected 1075 unique E3 ligases to achieve comprehensive coverage of E3 ligases for PROTAC, which systematically characterized E3 ligases from seven different aspects, including chemical ligandability, expression patterns, protein-protein interactions (PPI), structure availability, functional essentiality, cellular location, and PPI interface. | Disease, Gene |
| **TCMIO** | TCMIO is a comprehensive database of traditional Chinese medicine on immuno-oncology, which contains 13,403 prescription-TCM-relations, 32,847 TCM-ingredient-relations, and 41,527 ingredient-target-relations. | TCM Prescription, TCM CMM, Compound, Gene |
| **SoFDA** | SoFDA includes data on 319 TCM syndromes, 1610 related symptoms, 3955 associated genes, 8045 diseases, 8937 modern medicine symptoms, 10,273 related genes, and 1359 TCM formulas with their 1214 herbs and 1796 target genes. It aims at bridges the gap between TCM syndromes and modern molecular biology. | TCM Prescription, TCM CMM, Compound |
| **ITCM** | ITCM is a user-friendly information platform that extensively curates mass scientific research data related to traditional Chinese medicine (TCM) and include three main modules, TCM literature (CMTP) MODULE, TCM small molecule expression profile (SMEP) module and TOOL module. | TCM CMM, Compound, Gene |
| **SymMap** | SymMap integrates traditional Chinese medicine (TCM) with modern medicine (MM) through both internal molecular mechanism and external symptom mapping. | TCM CMM, Compound, TCM Syndrome, TCM Symptom |
| **HERB** | HERB is a high-throughput experiment- and reference-guided database of traditional Chinese medicine, which has manually curated 1,241 gene targets and 494 modern disease information from PubMed references published in recent 10 years. | TCM CMM, Compound, Gene |
| **ccTCM** | ccTCM is a web-based platform designed to provide a component and compound-content-based resource on TCM and analysis services for medical experts. It contains 273 Chinese medicinal materials commonly used in clinical settings, covering 29 functional classifications. | TCM CMM, Compound |
| **DCABM-TCM** | DCABM-TCM is a database of constituents absorbed into blood and metabolites of traditional Chinese medicine. In current DCABM-TCM, 1816 blood constituents with chemical structures of 194 herbs and 192 prescriptions were manually mined from literature. In addition, 1893 blood constituents without structures were also been recorded in the DCABM-TCM as they were given in the source publications. | TCM CMM, Compound |
| **TCMSTD** | TCMSTD is a systematic analysis of the traditional Chinese medicine system toxicology database, which consists of 252 herbs, 22 formulas, 2,425 targets, 4,361 chemistry ingredients and 226 toxicity ingredients. | TCM CMM, Compound |
| **BATMAN-TCM 2.0** | BATMAN-TCM is an enhanced integrative database for known and predicted interactions between traditional Chinese medicine ingredients and target proteins. | TCM CMM, Compound |
| **NPACT** | NPACT is a curated database of Plant derived natural compounds that exhibit anti-cancerous activity. It contains 1574 entries and each record provides information on their structure, properties (physical, elemental and topological), cancer type, cell lines, inhibitory values (IC_50_, ED_50_, EC_50_, GI_50_), molecular targets, commercial suppliers and drug likeness of compounds. | Compound, Gene |
| **HIT 2.0** | HIT is a comprehensive and fully curated database to complement available resources on protein targets for FDA-approved drugs as well as the promising precursors. The 2.0 version hosts 10 031 compound-target activity pairs with quality indicators between 2208 targets and 1237 ingredients from more than 1250 reputable herbs. | TCM CMM, Compound, Gene |

*^a^* The entity types are used in building TarKG.

**Table S3**. Brief descriptions of the six KGE algorithms.

| **Method** | **Entity Embedding** | **Relation Embedding** | **Score Function** | **Features** |
| --- | --- | --- | --- | --- |
| TransE | $h,t\in\mathbb{R}^{d}$ | $r\in\mathbb{R}^{d}$ | $-\parallel h+r-t\parallel$ | The model is simple and the training speed is fast, but it is only suitable for handling 1-to-1 relationships. |
| TransR | $h,t\in\mathbb{R}^{d}$ | $r\in\mathbb{R}^{k}$,  $M_{r}\mathbb{\in R}^{k\times d}$ | $-\parallel M_{r}h+r-M_{r}t\parallel_{2}^{2}$ | Different entity spaces are established to learn no 1-to-1 relationships, but the model complexity is too high and the training speed is slow. |
| RESCAL | $h,t\in\mathbb{R}^{d}$ | $M_{r}\in\mathbb{R}^{d\times d}$ | $h^{T}M_{r}t$ | It is a bilinear model with tensor decomposition as the main idea and high computational complexity. |
| DistMult | $h,t\in\mathbb{R}^{d}$ | $r\in\mathbb{R}^{d}$ | $h^{T}diag\left( r \right)t$ | The model limits the relationship matrix of the bilinear model to a diagonal matrix, which simplifies RESCAL, but can only handle symmetric relationships. |
| ComplEx | $h,t\in\mathbb{C}^{d}$ | $r\in\mathbb{C}^{d}$ | $h^{T}Re(diag\left( r \right)t)$ | This model introduces complex-valued embeddings, which can handle asymmetric relationships without excessive complexity. |
| RotatE | $h,t\in\mathbb{C}^{d}$ | $r\in\mathbb{C}^{d}$ | $\parallel h\circ r-t\parallel$ | It treats relationships as selections from head to tail entities, supporting three types of complex relationships: symmetric/asymmetric, inverse relationships, and relationship combinations. |

**Table S4**. The optimal hyperparameters of each KGE model on TarKG.

| Hyperparameters^a^ | Search range | KGE models | | | | | |
| --- | --- | --- | --- | --- | --- | --- | --- |
|  |  | TransE(L2) | TransR | RESCAL | DistMult | ComplEx | RotatE |
| batch_size | 1024 | 1024 | 1024 | 1024 | 1024 | 1024 | 1024 |
| neg_sample_size | 256 | 256 | 256 | 256 | 256 | 256 | 256 |
| batch_size_eval | 1000 | 1000 | 1000 | 1000 | 1000 | 1000 | 1000 |
| neg_sample_size_eval | 1000 | 1000 | 1000 | 1000 | 1000 | 1000 | 1000 |
| max_step | --- | 300000 | 300000 | 240000 | 240000 | 240000 | 300000 |
| lr | [0.005, 0.01, 0.1] | 0.25 | 0.1 | 0.1 | 0.25 | 0.1 | 0.01 |
| hidden_dim | [200, 400] | 600 | 200 | 200 | 600 | 600 | 600 |
| regularization_coef | [1E-9, 1E-7] | 1.00E-07 | 1.00E-05 | 1.00E-07 | 1.00E-07 | 1.00E-07 | 1.00E-07 |
| gamma | [8, 12, 16] | 12 | 12 | 12 | 8 | 12 | 12 |

^a^Abbreviation definition: **batch_size**, the batch size for training; **neg_sample_size**, the number of negative samples for each positive sample in the training; **batch_size_eval**, the batch size used for validation and test; **neg_sample_size_eval**, the number of negative samples for evaluating a positive sample; **max_step**, the maximal number of steps to train a model; **lr**, the learning rate; **hidden_dim**: the embedding size of relations and entities; **regularization_coef**, the coefficient for regularization; **gamma**, the margin value in the score function.

**Table S5**. The type and number of relationships contained by each entity pair in the TarKG.

| Entity Pair (No. of Relation Types) | Relation Type | Count | KG List |
| --- | --- | --- | --- |
| Anatomy-Anatomy (2) | is a | 23,675 | OpenBioLink |
|  | part of | 9,669 | OpenBioLink |
| Anatomy-Gene (7) | expresses | 1,642,013 | OpenBioLink, Hetionet, DRKG |
|  | expression present | 1,409,754 | PrimeKG |
|  | overexpression | 132,455 | OpenBioLink |
|  | underexpression | 128,125 | OpenBioLink |
|  | downregulates | 101,868 | Hetionet, DRKG |
|  | upregulates | 97,386 | Hetionet, DRKG |
|  | expression absent | 18,031 | PrimeKG |
| Biological_Process-Biological_Process (5) | is a | 55,983 | addKG, OpenBioLink |
|  | part of | 5,181 | OpenBioLink, addKG |
|  | regulates | 3,103 | addKG |
|  | negatively regulates | 2,693 | addKG |
|  | positively regulates | 2,685 | addKG |
| Cellular_Component-Cellular_Component (2) | is a | 5,177 | addKG, OpenBioLink |
|  | part of | 1,995 | addKG, OpenBioLink |
| Compound-Compound (7) | ddi | 1,296,400 | addKG, DRKG, BioKG, PharmKG |
|  | synergistic interaction | 1,194,662 | PrimeKG, addKG |
|  | associated with | 474,226 | addKG, PharmKG |
|  | negative correlate | 358,255 | addKG |
|  | positive correlate | 208,262 | addKG |
|  | compare | 77,509 | addKG |
|  | resembles | 6,411 | Hetionet, DRKG |
| Compound-Disease (10) | treats | 914,273 | addKG, PharmKG, DRKG, Hetionet |
|  | side effect | 532,723 | addKG, PharmKG, DRKG |
|  | associated with | 419,639 | BioKG, addKG, MSI, PharmKG |
|  | indication | 54,142 | PrimeKG, addKG, OpenBioLink, BioKG |
|  | contraindication | 22,965 | PrimeKG |
|  | alleviates | 8,731 | PharmKG, Hetionet, DRKG |
|  | role in pathogenesis | 3,619 | PharmKG, DRKG |
|  | inhibits cell growth | 3,229 | PharmKG, DRKG |
|  | off-label use | 2,029 | PrimeKG |
|  | biomarkers | 801 | PharmKG, DRKG |
| Compound-Gene (45) | binds | 981,131 | Hetionet, OpenBioLink, DRKG, addKG, PharmKG |
|  | functional | 821,183 | addKG |
|  | negative correlate | 568,192 | addKG |
|  | associated with | 530,835 | addKG, DRKG |
|  | positive correlate | 422,459 | addKG |
|  | transcriptome | 290,969 | tcmKG |
|  | interacts with | 207,434 | OpenBioLink, PharmKG, MSI, BioKG, addKG, DRKG |
|  | target | 106,457 | addKG, PrimeKG, DRKG, BioKG, tcmKG |
|  | E3 ligand | 43,483 | addKG |
|  | inhibits | 41,133 | OpenBioLink, DRKG, addKG, PharmKG |
|  | adme | 36,646 | addKG |
|  | affects expression | 32,485 | DRKG, PharmKG |
|  | catalysis | 23,634 | OpenBioLink |
|  | downregulates | 23,283 | Hetionet, DRKG, addKG |
|  | upregulates | 20,227 | DRKG, Hetionet |
|  | reaction | 18,922 | OpenBioLink |
|  | metabolism | 15,445 | PharmKG, DRKG, addKG |
|  | activates | 13,685 | OpenBioLink, addKG, DRKG |
|  | other | 9,617 | DRKG, addKG |
|  | enzyme | 5,715 | addKG, PrimeKG, DRKG, BioKG |
|  | channels | 4,610 | DRKG, PharmKG |
|  | agonism | 4,171 | DRKG, addKG |
|  | antagonism | 4,136 | addKG, DRKG |
|  | induces | 3,604 | addKG |
|  | transporter | 3,263 | addKG, PrimeKG, BioKG |
|  | enzyme activity | 2,280 | PharmKG, DRKG |
|  | blocker | 1,264 | DRKG, addKG |
|  | carrier | 935 | addKG, PrimeKG, DRKG, BioKG |
|  | toxicity | 697 | addKG |
|  | positive allosteric modulator | 627 | DRKG, addKG |
|  | agonism or antagonism | 397 | PharmKG |
|  | allosteric modulator | 311 | DRKG, addKG |
|  | modulator | 250 | addKG, DRKG |
|  | physical association | 203 | DRKG |
|  | potentiator | 132 | addKG |
|  | partial agonist | 104 | addKG, DRKG |
|  | ligand | 65 | addKG |
|  | substrate | 35 | addKG |
|  | opener | 34 | addKG |
|  | releasing agent | 24 | addKG |
|  | cofactor | 19 | addKG |
|  | inverse agonism | 17 | addKG |
|  | negative modulator | 15 | addKG |
|  | stabiliser | 12 | addKG |
|  | negative allosteric modulator | 11 | addKG |
| Compound-Pathway (1) | associated with | 3,762 | addKG, BioKG |
| Compound-Phenotype (1) | side effect | 106,729 | OpenBioLink, PrimeKG |
| Compound-Side_Effect (1) | causes | 148,938 | Hetionet, DRKG, BioKG |
| Disease-Anatomy (1) | localizes | 4,698 | Hetionet, DRKG |
| Disease-Disease (4) | is a | 49,106 | addKG, OpenBioLink |
|  | resembles | 761 | Hetionet, DRKG |
|  | associated with | 708 | PharmKG |
|  | ancestors | 491 | PharmKG |
| Disease-Gene (6) | associated with | 1,723,231 | addKG, PrimeKG, PharmKG, BioKG, Hetionet, DRKG, MSI, OpenBioLink |
|  | stimulates | 264,814 | addKG |
|  | inhibits | 202,170 | addKG |
|  | possible therapeutic effect | 11,092 | DRKG, PharmKG |
|  | upregulates | 9,103 | Hetionet, DRKG |
|  | downregulates | 8,866 | Hetionet, DRKG |
| Disease-Pathway (1) | associated with | 1,614 | BioKG |
| Disease-Phenotype (3) | phenotype present | 106,351 | PrimeKG |
|  | associated with | 23,618 | OpenBioLink |
|  | phenotype absent | 658 | PrimeKG |
| Disease-Symptom (1) | presents | 4,227 | Hetionet, DRKG |
| Gene-Biological_Process (1) | participates | 996,493 | Hetionet, DRKG, PrimeKG, BioKG, OpenBioLink, MSI |
| Gene-Cellular_Component (1) | participates | 400,070 | Hetionet, DRKG, BioKG, PrimeKG, OpenBioLink |
| Gene-Disease (11) | improper regulation linked to disease | 38,549 | DRKG |
|  | role in pathogenesis, or promotes progression | 30,610 | PharmKG |
|  | role in pathogenesis | 27,350 | DRKG |
|  | E3 highexpression | 19,899 | addKG |
|  | mutations affect, or polymorphisms alter risk | 14,668 | PharmKG, DRKG |
|  | causal mutations | 5,701 | DRKG |
|  | drug targets | 2,712 | PharmKG, DRKG |
|  | promotes progression | 1,776 | DRKG |
|  | polymorphisms alter risk | 1,716 | DRKG |
|  | overexpression | 1,051 | DRKG |
|  | biomarkers | 1,044 | DRKG |
| Gene-Gene (34) | ubiquitination | 1,152,275 | addKG, DRKG |
|  | ppi | 929,633 | OpenBioLink, addKG, Hetionet, DRKG, MSI, BioKG, PrimeKG, PharmKG |
|  | associated with | 839,131 | addKG, DRKG, PharmKG |
|  | reaction | 400,777 | OpenBioLink, DRKG |
|  | positive correlate | 394,823 | addKG |
|  | catalysis | 343,772 | OpenBioLink, DRKG |
|  | binds | 335,829 | PharmKG, OpenBioLink, DRKG |
|  | other | 302,335 | DRKG |
|  | regulates | 275,774 | Hetionet, DRKG |
|  | negative correlate | 215,738 | addKG |
|  | physical association | 127,090 | DRKG |
|  | activates | 121,706 | DRKG, PharmKG, OpenBioLink |
|  | covaries | 61,218 | Hetionet, DRKG |
|  | production by cell population | 45,506 | PharmKG, DRKG |
|  | relationships involving regulation and pathways | 43,839 | PharmKG |
|  | inhibits | 28,873 | OpenBioLink, DRKG |
|  | affects expression | 28,742 | PharmKG, DRKG |
|  | ptmod | 14,993 | DRKG |
|  | increases expression | 9,410 | DRKG |
|  | postTranslationalModifications | 5,032 | OpenBioLink |
|  | signaling pathway | 4,588 | DRKG |
|  | colocalization | 3,451 | DRKG |
|  | same protein or complex | 2,155 | DRKG |
|  | phosphorylation reaction | 1,324 | DRKG |
|  | host microbiome interaction | 869 | addKG |
|  | expresses | 756 | DRKG, OpenBioLink |
|  | transcription factor regulation | 347 | addKG |
|  | dephosphorylation reaction | 303 | DRKG |
|  | enhances response | 224 | DRKG |
|  | cleavage reaction | 93 | DRKG |
|  | oligomerization | 87 | addKG |
|  | histone modification | 85 | addKG |
|  | protein cleavage | 67 | DRKG |
|  | adp ribosylation reaction | 57 | DRKG |
| Gene-Molecular_Function (1) | participates | 346,409 | OpenBioLink, BioKG, Hetionet, DRKG, PrimeKG, MSI |
| Gene-Pathway (2) | participates | 169,094 | Hetionet, DRKG, BioKG |
|  | associated with | 162,857 | OpenBioLink, BioKG, PrimeKG |
| Gene-Phenotype (1) | associated with | 160,692 | OpenBioLink, PrimeKG |
| Molecular_Function-Molecular_Function (2) | is a | 14,660 | addKG, OpenBioLink |
|  | part of | 11 | addKG, OpenBioLink |
| Pathway-Biological_Process (1) | participates | 1,172 | BioKG |
| Pathway-Cellular_Component (1) | participates | 15,361 | BioKG |
| Pathway-Molecular_Function (1) | participates | 2,114 | BioKG |
| Pathway-Pathway (3) | complex in pathway | 20,632 | BioKG |
|  | complex top level pathway | 15,299 | BioKG |
|  | is a | 2,445 | BioKG |
| Phenotype-Phenotype (1) | is a | 18,416 | OpenBioLink |
| Symptom-Disease (1) | associated with | 42,755 | tcmKG |
| TCM_CMM-Compound (1) | includes | 124,806 | tcmKG |
| TCM_CMM-Disease (1) | associated with | 1,612,380 | tcmKG |
| TCM_CMM-Gene (1) | associated with | 5,812,852 | tcmKG |
| TCM_CMM-TCM_Symptom (1) | associated with | 21,476 | tcmKG |
| TCM_CMM-TCM_Syndrome (1) | associated with | 2,104 | tcmKG |
| TCM_Prescription-Disease (1) | associated with | 61,052 | tcmKG |
| TCM_Prescription-TCM_CMM (1) | includes | 24,375 | tcmKG |
| TCM_Prescription-TCM_Symptom (1) | associated with | 155 | tcmKG |
| TCM_Prescription-TCM_Syndrome(1) | associated with | 275 | tcmKG |
| TCM_Symptom-Disease (1) | associated with | 378,903 | tcmKG |
| TCM_Symptom-Symptom (1) | associated with | 3,231 | tcmKG |
| TCM_Symptom-TCM_Syndrome (1) | associated with | 9,260 | tcmKG |
| TCM_Syndrome-Disease (1) | associated with | 422,383 | tcmKG |

**Table S6**. Comparation of TarKG with other biomedical knowledge graphs.

| **KG Dataset** | **Design Usage** | **No. of Entities** | **No. of Triples** | **No. of Entity Types** | **No. of Relation Types** | **Last Update** |
| --- | --- | --- | --- | --- | --- | --- |
| **Hetionet** | Repurposing | 47K | 2.2M | 11 | 24 | 2017 |
| **PharmKG** | Repurposing/Target Prediction | 7.6K | 0.5M | 3 | 29 | 2020 |
| **OpenBioLink** | Benchmark | 184K | 4.7M | 7 | 30 | 2020 |
| **BioKG** | Benchmark | 105K | 2M | 10 | 17 | 2020 |
| **DRKG** | Repurposing | 97K | 5.7M | 13 | 107 | 2020 |
| **MSI network** | Disease Treatment Mechanisms | 30K | 0.5M | 4 | 6 | 2021 |
| **PrimeKG** | Personalized Medicine | 129K | 8M | 10 | 30 | 2023 |
| **mergeKG^a^** | - | 326K | 15.3M | 11 | 140 | Now |
| **TarKG** | Disease Target Discovery | 1143K | 32.8M | 15 | 171 | Now |

**^a^** The results of merging the existing knowledge graphs in the table.

**Table S7**. The number of relations of different entity pairs in TarKG.

| **Entity Pair** | **Relation Type Number** | **Count** | **Percent** |
| --- | --- | --- | --- |
| TCM_CMM-Gene | 1 | 5,812,852 | 17.72% |
| Gene-Gene | 34 | 5,690,902 | 17.35% |
| Compound-Gene | 45 | 4,240,151 | 12.92% |
| Compound-Compound | 7 | 3,615,725 | 11.02% |
| Anatomy-Gene | 7 | 3,529,632 | 10.76% |
| Disease-Gene | 6 | 2,219,276 | 6.76% |
| Compound-Disease | 10 | 1,962,151 | 5.98% |
| TCM_CMM-Disease | 1 | 1,612,380 | 4.91% |
| Gene-Biological_Process | 1 | 996,493 | 3.04% |
| TCM_Syndrome-Disease | 1 | 422,383 | 1.29% |
| Gene-Cellular_Component | 1 | 400,070 | 1.22% |
| TCM_Symptom-Disease | 1 | 378,903 | 1.15% |
| Gene-Molecular_Function | 1 | 346,409 | 1.06% |
| Gene-Pathway | 2 | 331,951 | 1.01% |
| Gene-Phenotype | 1 | 160,692 | 0.49% |
| Compound-Side_Effect | 1 | 148,938 | 0.45% |
| Gene-Disease | 11 | 145,076 | 0.44% |
| Disease-Phenotype | 3 | 130,627 | 0.40% |
| TCM_CMM-Compound | 1 | 124,806 | 0.38% |
| Compound-Phenotype | 1 | 106,729 | 0.33% |
| Biological_Process-Biological_Process | 5 | 69,645 | 0.21% |
| TCM_Prescription-Disease | 1 | 61,052 | 0.19% |
| Disease-Disease | 4 | 51,066 | 0.16% |
| Symptom-Disease | 1 | 42,755 | 0.13% |
| Pathway-Pathway | 3 | 38,376 | 0.12% |
| Anatomy-Anatomy | 2 | 33,344 | 0.10% |
| TCM_Prescription-TCM_CMM | 1 | 24,375 | 0.07% |
| TCM_CMM-TCM_Symptom | 1 | 21,476 | 0.07% |
| Phenotype-Phenotype | 1 | 18,416 | 0.06% |
| Pathway-Cellular_Component | 1 | 15,361 | 0.05% |
| Molecular_Function-Molecular_Function | 2 | 14,671 | 0.04% |
| TCM_Symptom-TCM_Syndrome | 1 | 9,260 | 0.03% |
| Cellular_Component-Cellular_Component | 2 | 7,172 | 0.02% |
| Disease-Anatomy | 1 | 4,698 | 0.01% |
| Disease-Symptom | 1 | 4,227 | 0.01% |
| Compound-Pathway | 1 | 3,762 | 0.01% |
| TCM_Symptom-Symptom | 1 | 3,231 | 0.01% |
| Pathway-Molecular_Function | 1 | 2,114 | 0.01% |
| TCM_CMM-TCM_Syndrome | 1 | 2,104 | 0.01% |
| Disease-Pathway | 1 | 1,614 | 0.00% |
| Pathway-Biological_Process | 1 | 1,172 | 0.00% |
| TCM_Prescription-TCM_Syndrome | 1 | 275 | 0.00% |
| TCM_Prescription-TCM_Symptom | 1 | 155 | 0.00% |
| Total | 171 | 32,806,467 | 100.00% |

**Table S8**. The number and proportion of relationships involving entities in TarKG.

| **Entity** | **Count** | **Percent** |
| --- | --- | --- |
| Gene | 29,564,406 | 45.06% |
| Compound | 13,817,987 | 21.06% |
| TCM_CMM | 7,597,993 | 11.58% |
| Disease | 7,087,274 | 10.80% |
| Anatomy | 3,601,018 | 5.49% |
| Biological_Process | 1,136,955 | 1.73% |
| Phenotype | 434,880 | 0.66% |
| TCM_Syndrome | 434,022 | 0.66% |
| Pathway | 432,726 | 0.66% |
| Cellular_Component | 429,775 | 0.66% |
| TCM_Symptom | 413,025 | 0.63% |
| Molecular_Function | 377,865 | 0.58% |
| Side_Effect | 148,938 | 0.23% |
| TCM_Prescription | 85,857 | 0.13% |
| Symptom | 50,213 | 0.08% |

**Table S9**. List of the top 10 potential protein targets for AD with different KGE models.

| Rank | TransE (L2) | RESCAL | DistMult | ComplEx | RotatE |
| --- | --- | --- | --- | --- | --- |
| 1 | Q15831 | **Q92934** | **Q92934** | Q13164 | **Q12809** |
| 2 | O75030 | P47989 | O14649 | P24666 | Q9NUW8 |
| 3 | **Q92934** | O75907 | Q15139 | P17342 | Q03164 |
| 4 | P06280 | P23280 | **Q12809** | O14656 | Q9Y5Y4 |
| 5 | P16233 | P51812 | P02144 | **Q12809** | P10253 |
| 6 | P49327 | P46059 | Q13164 | P10912 | P15428 |
| 7 | P19784 | Q16790 | Q96RD7 | P09848 | P06280 |
| 8 | Q13489 | O95271 | P47989 | P11684 | P51151 |
| 9 | P08034 | P49327 | Q09470 | P10721 | P56373 |
| 10 | P50336 | Q96PN6 | Q8IU80 | P41240 | B2RXH2 |

**Table S10.** Literature analysis of the top 10 potential protein targets for AD predicted with RESCAL.

| Rank | Target ID | Target Name | Evidence type | Literature Summary |
| --- | --- | --- | --- | --- |
| 1 | Q92934 | BAD_HUMAN | Direct evidence | (1) Apoptosis is one of the causes of AD development (Obulesu and Lakshmi 2014).  (2) The Bcl-2 family member BAD promotes apoptosis and has been shown to be upregulated in AD brains (Kitamura et al. 1998). |
| 2 | P47989 | XDH_HUMAN | Potential associations | (1) Xanthine dehydrogenase (XDH) is a major producer of reactive oxygen species (ROS), and elevated ROS levels are a hallmark of AD (Pathak et al. 2019). |
| 3 | O75907 | DGAT1_HUMAN | Potentially associations | (1) Lipid metabolism plays an important role in the pathogenesis of AD (Yin 2023).  (2) Diacylglycerol acyltransferase 1 (DGAT1) is essential for lipid synthesis (Yang et al. 2022). |
| 4 | P23280 | CAH6_HUMAN | Potentially associations | (1) Several recent studies have suggested that carbonic anhydrases (CAs) could be potential therapeutic targets for AD (Provensi et al. 2019). |
| 5 | P51812 | KS6A3_HUMAN | Unknown | - |
| 6 | P46059 | S15A1_HUMAN | Potentially associations | (1) Solute carrier (SLC) transporters are susceptibility genes or pathogenic genes for various diseases, such as metabolic diseases and neurological dysfunction. GWAS meta-analysis identified SLC24A4 as a susceptibility locus for AD (Zhang et al. 2018). |
| 7 | Q16790 | CAH9_HUMAN | Potentially associations | (1) Several recent studies have suggested that carbonic anhydrases (CAs) could be potential therapeutic targets for AD (Provensi et al. 2019). |
| 8 | O95271 | TNKS1_HUMAN | Potentially associations | (1) Poly(ADP-ribose) polymerase (PARP) is involved in the pathogenesis of some neurodegenerative diseases, including AD (Lastra et al. 2007). |
| 9 | P49327 | FAS_HUMAN | Direct evidence | (1) Fatty acid synthase (FASN) inhibition protects against AD-related toxicities, suggesting it as a potential therapeutic target in AD (Ates et al. 2020). |
| 10 | Q96PN6 | ADCYA_HUMAN | Potentially associations | (1) Dysregulation of adenylyl cyclases (ADCYs) has been implicated in various neurological diseases, including AD and depression (Devasani and Yao 2022). |

**Table S11**. List of the top 10 of potentially related diseases for CSN5 with five KGE models.

| Rank | TransE (L2) | RESCAL | DistMult | ComplEx | RotatE |
| --- | --- | --- | --- | --- | --- |
| 1 | DOID:5041 | MONDO:1011331 | DOID:421 | DOID:2154 | DOID:0050736 |
| 2 | DOID:0060892 | DOID:12662 | MESH:D012678 | DOID:3911 | DOID:630 |
| 3 | MESH:D006223 | MONDO:0043346 | DOID:326 | DOID:7400 | DOID:0050735 |
| 4 | MESH:D000236 | MONDO:0006926 | DOID:615 | **MESH:D007951** | DOID:0080014 |
| 5 | DOID:5419 | MESH:D054218 | DOID:9252 | DOID:14175 | MESH:D000094602 |
| 6 | DOID:1037 | MONDO:0012474 | DOID:5119 | DOID:11476 | DOID:0060240 |
| 7 | MONDO:0021223 | DOID:3083 | DOID:557 | MESH:D012327 | MONDO:0020022 |
| 8 | DOID:6536 | MONDO:0014085 | DOID:2938 | DOID:9538 | DOID:0060551 |
| 9 | **MESH:D007951** | DOID:3331 | MESH:D010146 | MESH:D025064 | DOID:0050737 |
| 10 | DOID:3168 | DOID:14261 | DOID:5223 | DOID:12704 | DOID:14227 |

**Table S12.** Literature analysis of the top 10 potentially related diseases for CSN5 with RESCAL.

| Rank | Target ID | Target Name | Evidence Type | Literature Summary |
| --- | --- | --- | --- | --- |
| 1 | MONDO:1011331 | Metabolic Disease, Non-Human Animal | Unknown | - |
| 2 | DOID:12662 | Paracoccidioidomycosis | Unknown | - |
| 3 | MONDO:0043346 | Progressive Transformation of Germinal Centers | Potentially associations | (1) Jun activation domain-binding protein 1 (Jab1, also known as CSN5) is crucial for the expression of Bcl6, a transcription repressor required for germinal center formation (Sitte et al. 2012). |
| 4 | MONDO:0006926 | Haemophilus Infectious Disease | Potentially associations | (1) CSN5 is a type of deubiquitinase (DUB), and Haemophilus influenzae infection may be related to the ubiquitination process (Li and Zhong 2018). |
| 5 | MESH:D054218 | Precursor T-Cell Lymphoblastic Leukemia-Lymphoma | Potentially associations | (1) FBW7 (an E3 ligase) suppresses adult T-cell leukemia-lymphoma progression by inhibiting the Notch signaling pathway, which is possibly associated with the ubiquitination process (Fhu and Ali 2021). |
| 6 | MONDO:0012474 | Autosomal Dominant Nocturnal Frontal Lobe Epilepsy 4 | Potentially associations | (1) Jab1 activated by 5-HT6 receptors may play an important role in epileptogenesis and cognitive impairment (Liu et al. 2019).  (2) The Jab1/phospho-c-jun pathway under the control of 5-HT6 receptors plays a crucial role in epileptic status. In addition to its beneficial effects on cognition, 5-HT6 receptor blockade may be considered as a novel therapeutic strategy for epilepsy (Chaumont-Dubel et al. 2020). |
| 7 | DOID:3083 | Chronic Obstructive Pulmonary Disease | Potentially associations | (1) Apoptosis induced by the tumor suppressor protein p53 may be a cause of chronic obstructive pulmonary disease (COPD), and CSN5/Jab-1 has been shown to regulate the degradation of p53. Additionally, macrophage migration inhibitory factor (MIF) has been shown to physically interact with Jab1, and the combination of the two can antagonize p53 induction and apoptosis (Damico et al. 2011).  (2) Additional increases in MIF levels were observed in patients with acute COPD exacerbations (Günther et al. 2019). |
| 8 | MONDO:0014085 | Hydrocephalus, Nonsyndromic, Autosomal Recessive 2 | Unknown | - |
| 9 | DOID:3331 | Frontal Lobe Epilepsy | Potentially associations | (1) Jab1 activated by 5-HT6 receptors may play an important role in epileptogenesis and cognitive impairment (Liu et al. 2019).  (2) The Jab1/phospho-c-jun pathway under the control of 5-HT6 receptors plays a crucial role in epileptic status. In addition to its beneficial effects on cognition, 5-HT6 receptor blockade may be considered as a novel therapeutic strategy for epilepsy (Chaumont-Dubel et al. 2020). |
| 10 | DOID:14261 | Fragile X Syndrome | Potentially associations | (1) Serotonin (5-hydroxytryptamine, 5-HT), a crucial neurotransmitter during early neurodevelopment, is implicated in various neurodevelopmental disorders, including Fragile X syndrome. The 5-HT6 receptor (5-HT6R), one of the recently discovered serotonin receptors, has been shown to involve the Jun activation domain-binding protein 1 (Jab1) pathway in its signaling cascade (Lee et al. 2022).  (2) The mechanistic target of rapamycin (mTOR) pathway, a component of the ubiquitination system (UbS), is linked to the fragile X mental retardation protein (FMRP), the loss of which causes fragile X syndrome (Kasherman et al. 2020). |

**3. Supplementary Figures**

**
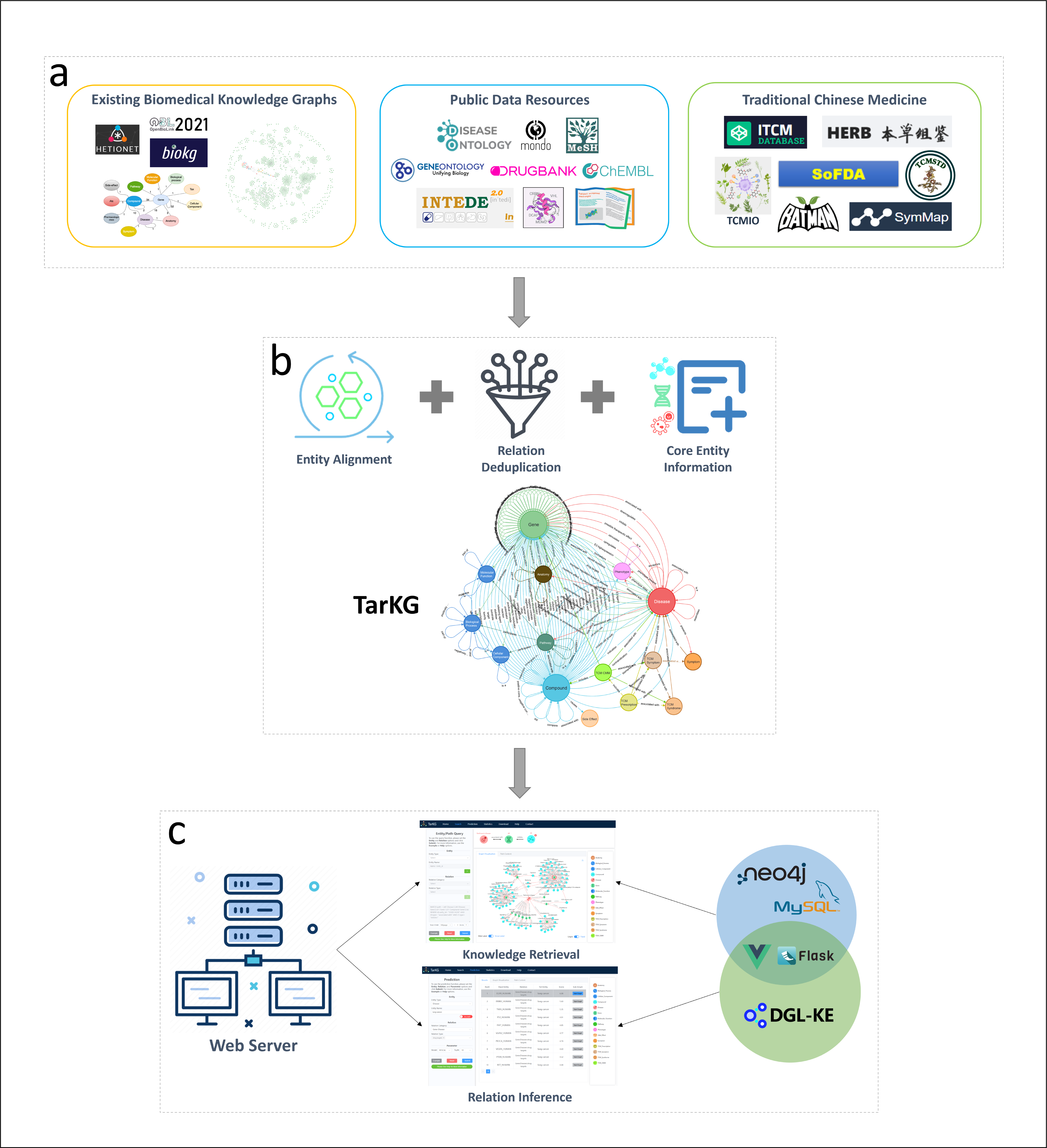
**

**Figure S1**. Pipeline for TarKG construction, web server and applications. (a) The main data components and sources of TarKG. (b) Data process of TarKG construction. (c) Web server and applications of TarKG.


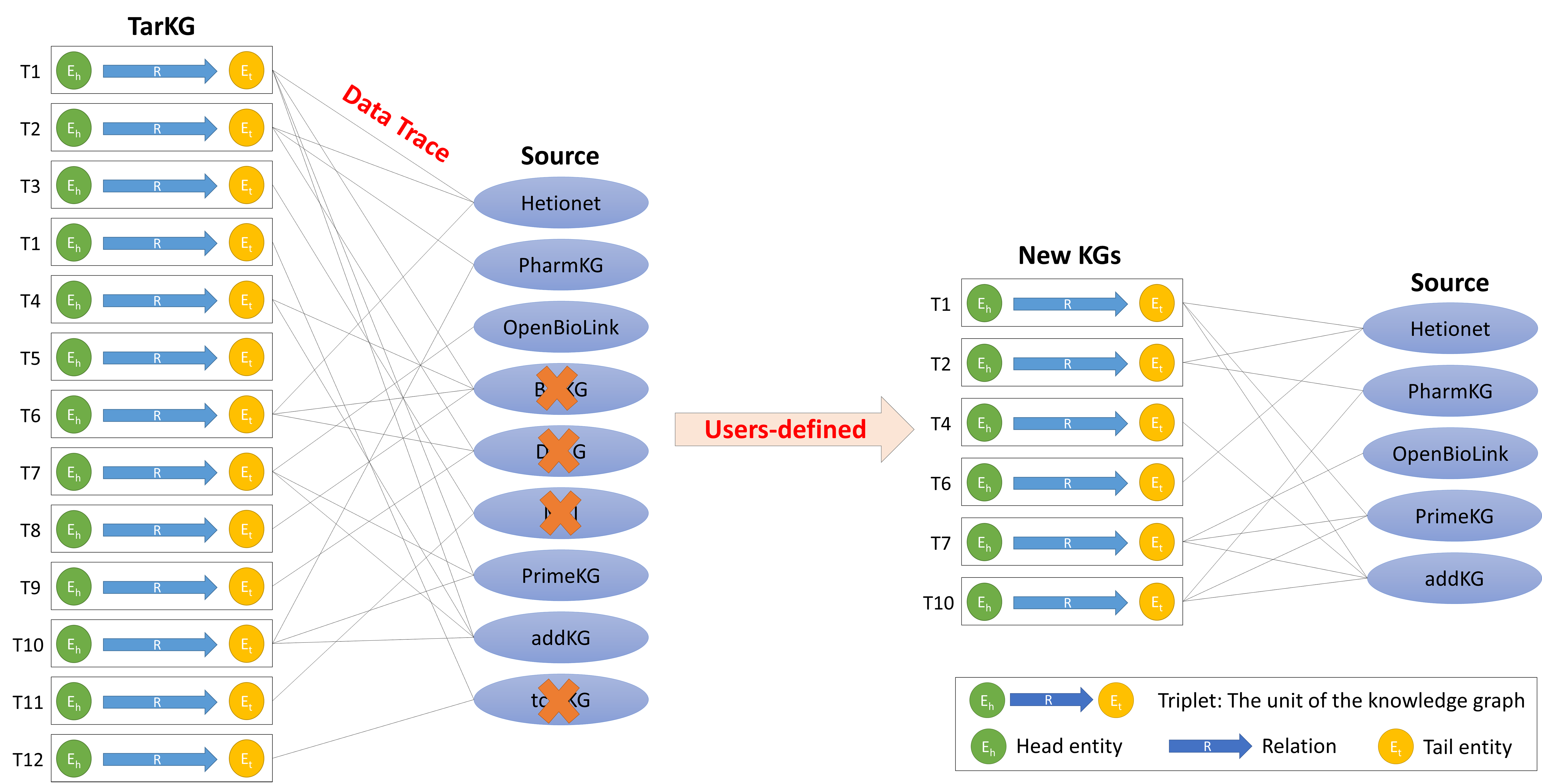


**Figure S2**. The mechanism sample graph of data tracing and knowledge graph reconstruction in TarKG.


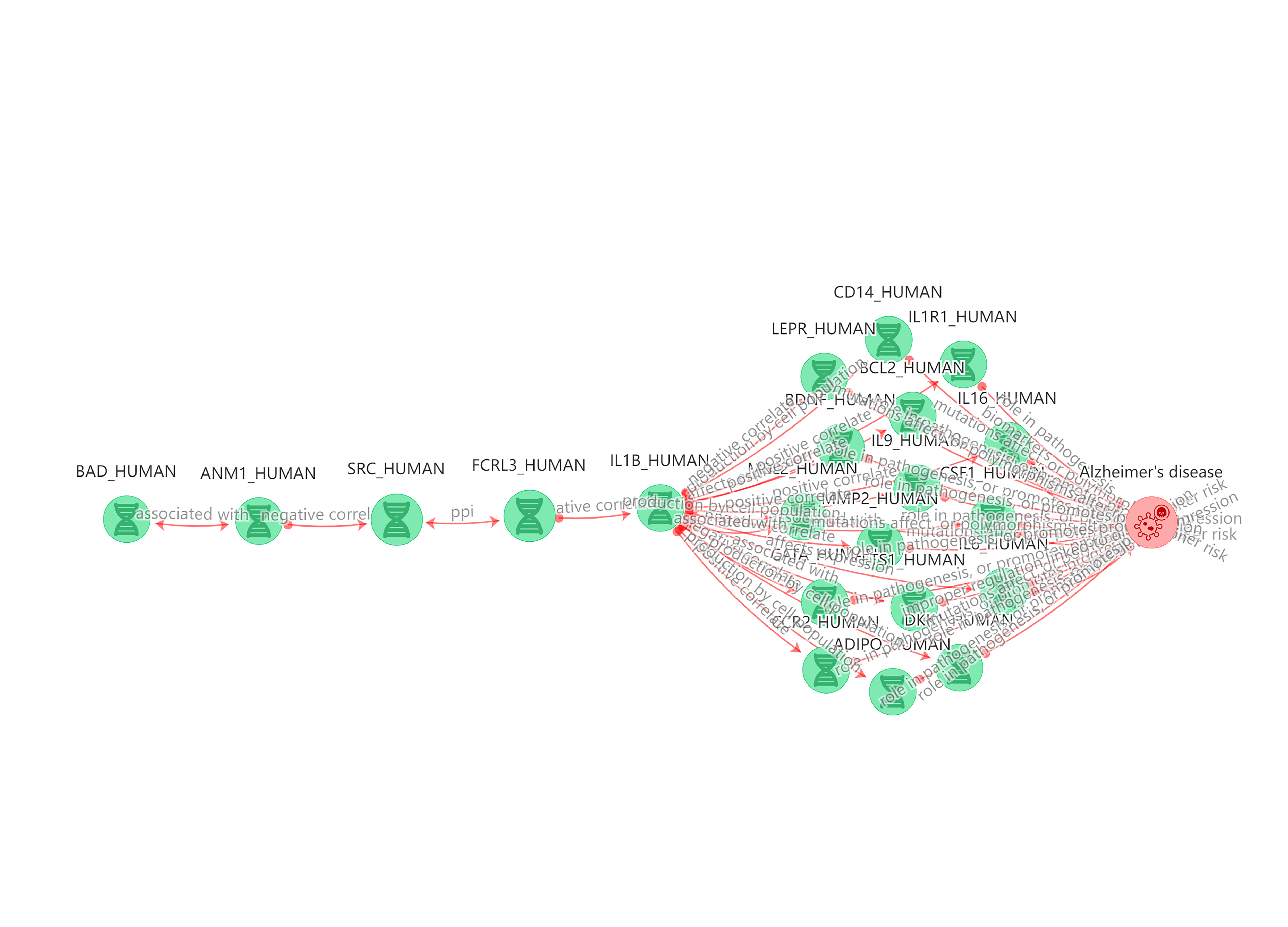


**Figure S3**. Graphical illustration of partial existing paths between BAD and AD in TarKG, with a path length of 6.

**Reference**

Agrawal A, Balcı H, Hanspers K *et al.* WikiPathways 2024: next generation pathway database. *Nucleic Acids Res* 2023;52(D1):D679-D689.

Amberger JS, Bocchini CA, Scott AF *et al.* OMIM.org: leveraging knowledge across phenotype–gene relationships. *Nucleic Acids Res* 2018;47(D1):D1038-D1043.

Ashburner M, Ball CA, Blake JA *et al.* Gene Ontology: tool for the unification of biology. *Nat Genet* 2000;25(1):25-29.

Ates G, Goldberg J, Currais A *et al.* CMS121, a fatty acid synthase inhibitor, protects against excess lipid peroxidation and inflammation and alleviates cognitive loss in a transgenic mouse model of Alzheimer's disease. *Redox Biol* 2020;36:101648.

Baron JA, Johnson CS-B, Schor MA *et al.* The DO-KB Knowledgebase: a 20-year journey developing the disease open science ecosystem. *Nucleic Acids Res* 2023;52(D1):D1305-D1314.

Bodenreider O. The Unified Medical Language System (UMLS): integrating biomedical terminology. *Nucleic Acids Res* 2004;32(suppl_1):D267-D270.

Cai C, Wu Q, Hong H *et al.* In silico identification of natural products from Traditional Chinese Medicine for cancer immunotherapy. *Sci Rep* 2021;11(1):3332.

Chaumont-Dubel S, Dupuy V, Bockaert J *et al.* The 5-HT6 receptor interactome: New insight in receptor signaling and its impact on brain physiology and pathologies. *Nucleic Acids Res* 2020;172:107839.

Consortium TU. UniProt: the Universal Protein Knowledgebase in 2023. *Nucleic Acids Res* 2022;51(D1):D523-D531.

Damico R, Simms T, Kim BS *et al.* p53 Mediates Cigarette Smoke–Induced Apoptosis of Pulmonary Endothelial Cells. *Am J Respir Cell Mol Biol* 2011;44(3):323-332.

Davis AP, Wiegers TC, Johnson RJ *et al.* Comparative Toxicogenomics Database (CTD): update 2023. *Nucleic Acids Res* 2022;51(D1):D1257-D1262.

Devasani K,Yao Y. Expression and functions of adenylyl cyclases in the CNS. *Fluids Barriers CNS* 2022;19(1):23.

Diehl AD, Meehan TF, Bradford YM *et al.* The Cell Ontology 2016: enhanced content, modularization, and ontology interoperability. *J Biomed Semantics* 2016;7(1):44.

Fang S, Dong L, Liu L *et al.* HERB: a high-throughput experiment- and reference-guided database of traditional Chinese medicine. *Nucleic Acids Res* 2020;49(D1):D1197-D1206.

Fhu CW, Ali A. Dysregulation of the Ubiquitin Proteasome System in Human Malignancies: A Window for Therapeutic Intervention. *Cancers* 2021;13(7):1513.

Gargano MA, Matentzoglu N, Coleman B *et al.* The Human Phenotype Ontology in 2024: phenotypes around the world. *Nucleic Acids Res* 2023;52(D1):D1333-D1346.

Günther S, Fagone P, Jalce G *et al.* Role of MIF and D-DT in immune-inflammatory, autoimmune, and chronic respiratory diseases: from pathogenic factors to therapeutic targets. *Drug Discov Today* 2019;24(2):428-439.

Haendel MA, Balhoff JP, Bastian FB *et al.* Unification of multi-species vertebrate anatomy ontologies for comparative biology in Uberon. *J Biomed Semantics* 2014;5(1):21.

Hastings J, Owen G, Dekker A *et al.* ChEBI in 2016: Improved services and an expanding collection of metabolites. *Nucleic Acids Res* 2015;44(D1):D1214-D1219.

Huang Y, Fang J, Lu W *et al.* A Systems Pharmacology Approach Uncovers Wogonoside as an Angiogenesis Inhibitor of Triple-Negative Breast Cancer by Targeting Hedgehog Signaling. *Cell Chem Biol* 2019;26(8):1143-1158.e1146.

Kanehisa M, Furumichi M, Sato Y *et al.* KEGG for taxonomy-based analysis of pathways and genomes. *Nucleic Acids Res* 2022;51(D1):D587-D592.

Kasherman MA, Premarathne S, Burne THJ *et al.* The Ubiquitin System: a Regulatory Hub for Intellectual Disability and Autism Spectrum Disorder. *Mol Neurobiol* 2020;57(5):2179-2193.

Kim S, Chen J, Cheng T *et al.* PubChem 2023 update. *Nucleic Acids Res* 2022;51(D1):D1373-D1380.

Kitamura Y, Shimohama S, Kamoshima W *et al.* Alteration of proteins regulating apoptosis, Bcl-2, Bcl-x, Bax, Bak, Bad, ICH-1 and CPP32, in Alzheimer's disease. *Brain Res* 1998;780(2):260-269.

Knox C, Wilson M, Klinger Christen M *et al.* DrugBank 6.0: the DrugBank Knowledgebase for 2024. *Nucleic Acids Res* 2023;52(D1):D1265-D1275.

Kong X, Liu C, Zhang Z *et al.* BATMAN-TCM 2.0: an enhanced integrative database for known and predicted interactions between traditional Chinese medicine ingredients and target proteins. *Nucleic Acids Res* 2023;52(D1):D1110-D1120.

Kuhn M, Letunic I, Jensen LJ *et al.* The SIDER database of drugs and side effects. *Nucleic Acids Res* 2015;44(D1):D1075-D1079.

Lastra CAdl, Villegas I,Sanchez-Fidalgo S. Poly(ADP-Ribose) Polymerase Inhibitors: New Pharmacological Functions and Potential Clinical Implications. *Curr Pharm Des* 2007;13(9):933-962.

Lee A, Choo H, Jeon B. Serotonin Receptors as Therapeutic Targets for Autism Spectrum Disorder Treatment. *Int J Mol Sci* 2022;23(12):6515.

Li K, Zhong B. Regulation of Cellular Antiviral Signaling by Modifications of Ubiquitin and Ubiquitin-like Molecules. *Immune Netw* 2018;18(1).

Lipscomb CE. Medical Subject Headings (MeSH). *Bull Med Libr Assoc* 2000;88(3):265-266.

Liu C, Wen Y, Huang H *et al.* Over-expression of 5-HT6 Receptor and Activated Jab-1/p-c-Jun Play Important Roles in Pilocarpine-Induced Seizures and Learning-Memory Impairment. *J Mol Neurosci* 2019;67(3):388-399.

Liu X, Liu J, Fu B *et al.* DCABM-TCM: A Database of Constituents Absorbed into the Blood and Metabolites of Traditional Chinese Medicine. *J Chem Inf Model* 2023;63(15):4948-4959.

Liu Z, Cai C, Du J *et al.* TCMIO: A Comprehensive Database of Traditional Chinese Medicine on Immuno-Oncology. *Front Pharmacol* 2020;11.

Maglott D, Ostell J, Pruitt KD *et al.* Entrez Gene: gene-centered information at NCBI. *Nucleic Acids Res* 2010;39(suppl_1):D52-D57.

Milacic M, Beavers D, Conley P *et al.* The Reactome Pathway Knowledgebase 2024. *Nucleic Acids Res* 2023;52(D1):D672-D678.

O'Boyle NM, Banck M, James CA *et al.* Open Babel: An open chemical toolbox. *J Cheminformatics* 2011;3(1):33.

Obulesu M, Lakshmi MJ. Apoptosis in Alzheimer’s Disease: An Understanding of the Physiology, Pathology and Therapeutic Avenues. *Neurochem Res* 2014;39(12):2301-2312.

Pathak GA, Silzer TK, Sun J *et al.* Genome-Wide Methylation of Mild Cognitive Impairment in Mexican Americans Highlights Genes Involved in Synaptic Transport, Alzheimer’s Disease-Precursor Phenotypes, and Metabolic Morbidities. *J Alzheimer's Dis* 2019;72:733-749.

Provensi G, Carta F, Nocentini A *et al.* A New Kid on the Block? Carbonic Anhydrases as Possible New Targets in Alzheimer’s Disease. *Int J Mol Sci* 2019;20(19):4724.

Rodchenkov I, Babur O, Luna A *et al.* Pathway Commons 2019 Update: integration, analysis and exploration of pathway data. *Nucleic Acids Res* 2019;48(D1):D489-D497.

Sitte S, Gläsner J, Jellusova J *et al.* JAB1 Is Essential for B Cell Development and Germinal Center Formation and Inversely Regulates Fas Ligand and Bcl6 Expression. *J Immunol* 2012;188(6):2677-2686.

Song L, Qian W, Yin H *et al.* TCMSTD 1.0: a systematic analysis of the traditional Chinese medicine system toxicology database. *Sci China Life Sci* 2023;66(9):2189-2192.

Tian S, Zhang J, Yuan S *et al.* Exploring pharmacological active ingredients of traditional Chinese medicine by pharmacotranscriptomic map in ITCM. *Brief Bioinformatics* 2023;24(2).

Vasilevsky NA, Matentzoglu NA, Toro S *et al.* Mondo: Unifying diseases for the world, by the world. *medRxiv* 2022:2022.2004.2013.22273750.

Wishart DS, Kruger R, Sivakumaran A *et al.* PathBank 2.0—the pathway database for model organism metabolomics. *Nucleic Acids Res* 2023;52(D1):D654-D662.

Wu Y, Zhang F, Yang K *et al.* SymMap: an integrative database of traditional Chinese medicine enhanced by symptom mapping. *Nucleic Acids Res* 2018;47(D1):D1110-D1117.

Yang D, Wang X, Zhang L *et al.* Lipid metabolism and storage in neuroglia: role in brain development and neurodegenerative diseases. *Cell Biosci* 2022;12(1):106.

Yang D, Zhu Z, Yao Q *et al.* ccTCM: A quantitative component and compound platform for promoting the research of traditional Chinese medicine. *Comput Struct Biotechnol J* 2023;21:5807-5817.

Yin F. Lipid metabolism and Alzheimer's disease: clinical evidence, mechanistic link and therapeutic promise. *FEBS J* 2023;290(6):1420-1453.

Zdrazil B, Felix E, Hunter F *et al.* The ChEMBL Database in 2023: a drug discovery platform spanning multiple bioactivity data types and time periods. *Nucleic Acids Res* 2023;52(D1):D1180-D1192.

Zhang Y, Wang N, Du X *et al.* SoFDA: an integrated web platform from syndrome ontology to network-based evaluation of disease–syndrome–formula associations for precision medicine. *Sci Bull* 2022;67(11):1097-1101.

Zhang Y, Zhang Y, Sun K *et al.* The SLC transporter in nutrient and metabolic sensing, regulation, and drug development. *J Mol Cell Biol* 2018;11(1):1-13.
