# Supplementary figures and images for: TarKG: a comprehensive biomedical knowledge graph for target discovery
Source: Bioinformatics. 2024 Oct 11;40(10):btae598. doi: 10.1093/bioinformatics/btae598 (PMC11513019; doi:10.1093/bioinformatics/btae598)

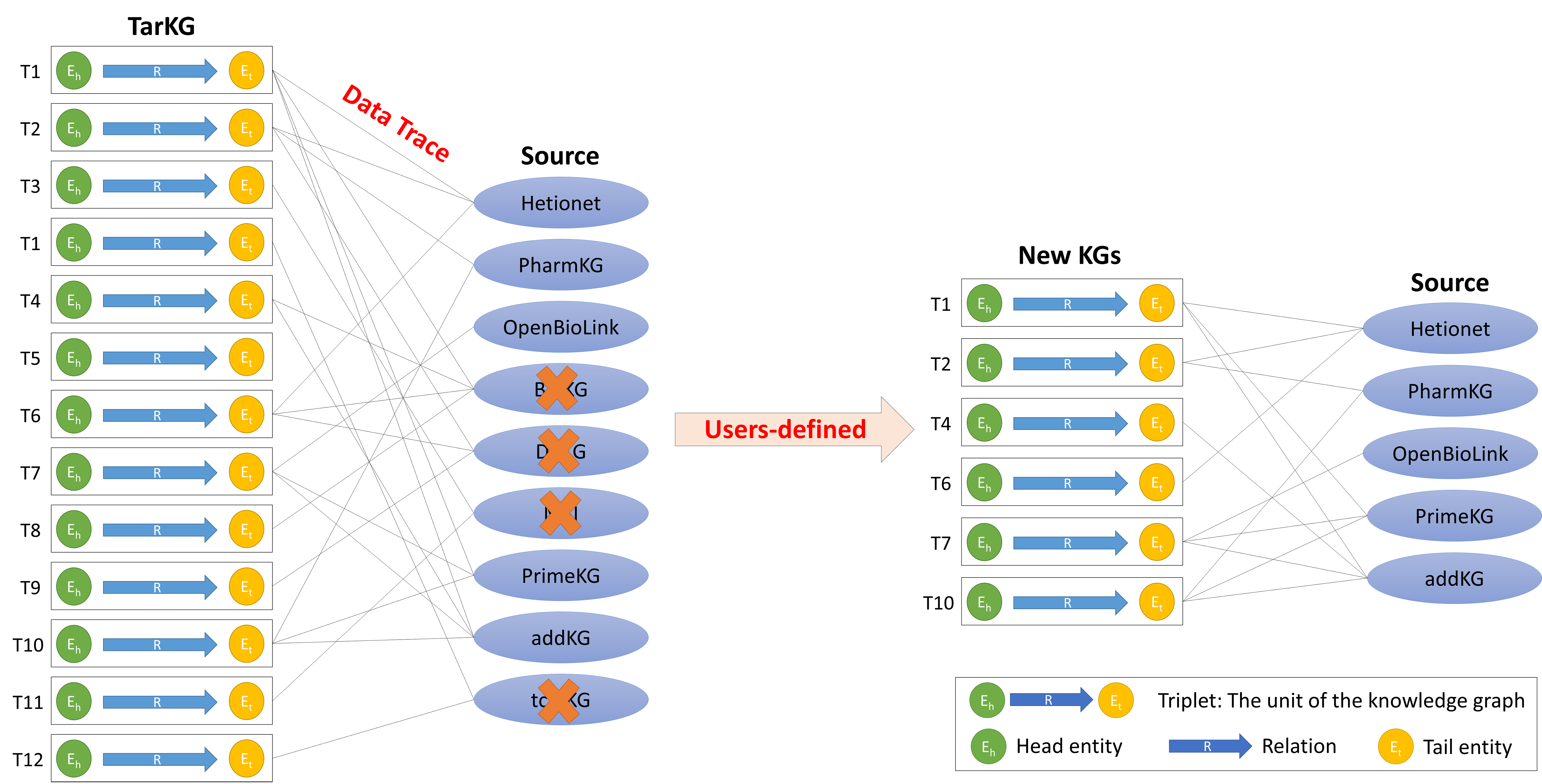

Supplement: btae598_Supplementary_Data [file btae598_supplementary_data.zip › Figure S2. The mechanism sample graph of data tracing and knowledge graph reconstruction in TarKG.tif]

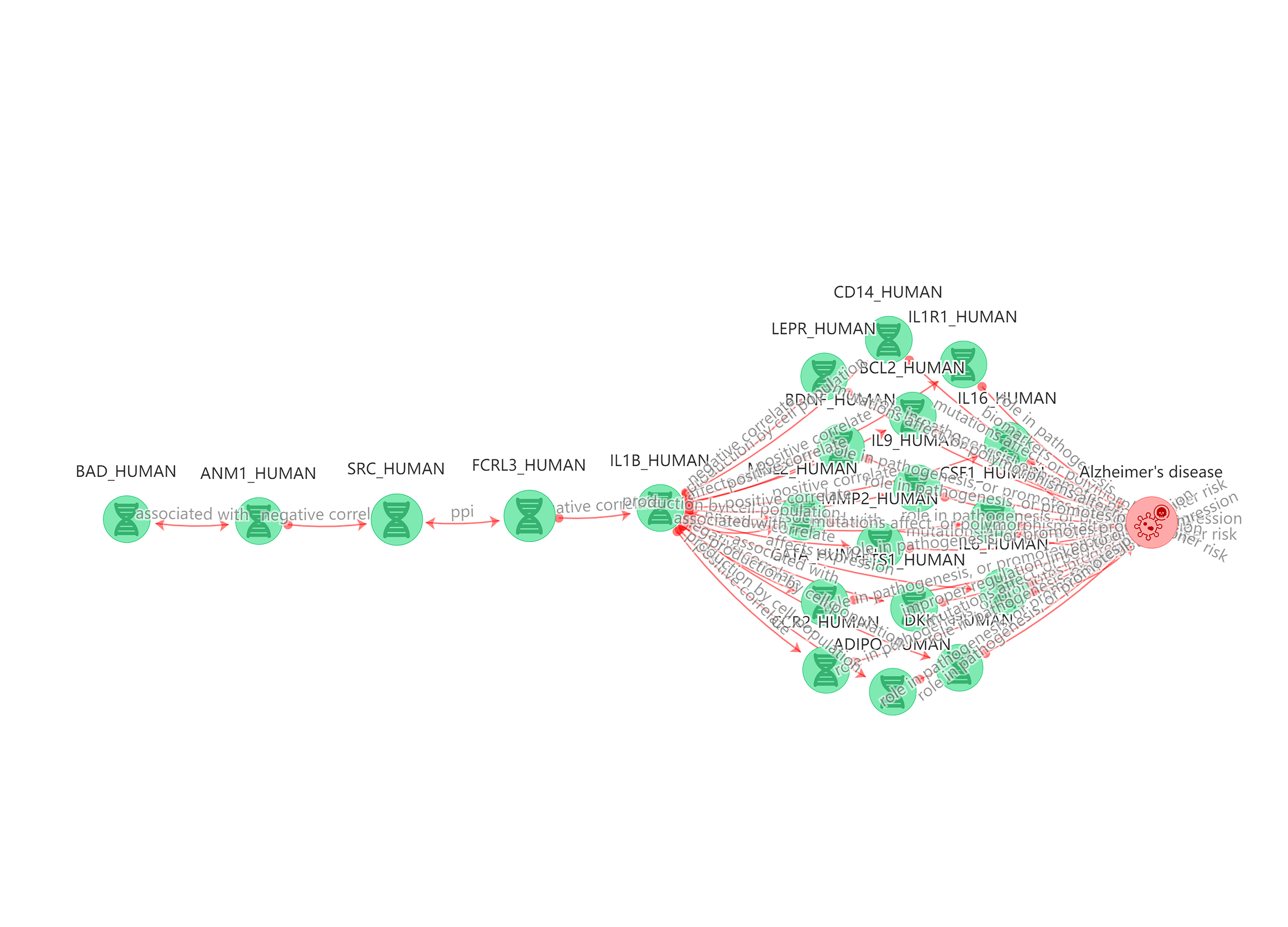

Supplement: btae598_Supplementary_Data [file btae598_supplementary_data.zip › 9.tif]

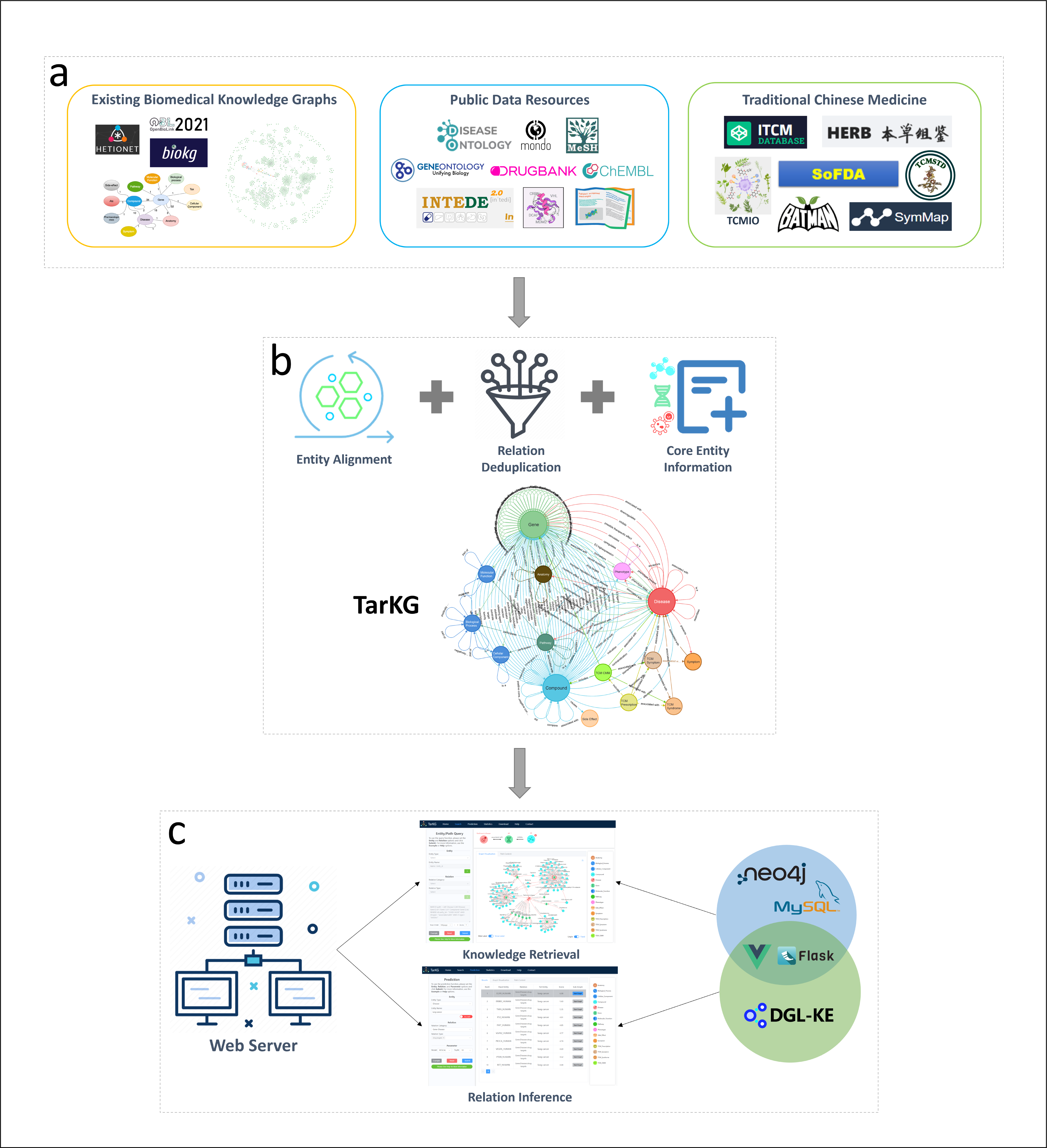

Supplement: btae598_Supplementary_Data [file btae598_supplementary_data.zip › Figure S1. Pipeline for TarKG construction, web server and applications.tif]
